# Conserved leaf–root metabolomic network asymmetry underpins divergent drought strategies

**DOI:** 10.1093/plcell/koag217

**Published:** 2026-07-15

**Authors:** Mirza Shoaib, Simone J Rochfort, Priyanka Reddy, Doris Ram, Matthew J Hayden, Surya Kant

**Affiliations:** Agriculture Victoria, Grains Innovation Park, 110 Natimuk Road, Horsham, Victoria 3400, Australia; School of Applied Systems Biology, La Trobe University, 5 Ring Road, Bundoora, Victoria 3083, Australia; School of Applied Systems Biology, La Trobe University, 5 Ring Road, Bundoora, Victoria 3083, Australia; Agriculture Victoria, AgriBio, Centre for AgriBioscience, 5 Ring Road, Bundoora, Victoria 3083, Australia; School of Chemistry, Bio21 Molecular Science and Biotechnology Institute, The University of Melbourne, 30 Flemington Road, Parkville, Victoria 3010, Australia; Agriculture Victoria, AgriBio, Centre for AgriBioscience, 5 Ring Road, Bundoora, Victoria 3083, Australia; School of Applied Systems Biology, La Trobe University, 5 Ring Road, Bundoora, Victoria 3083, Australia; Agriculture Victoria, AgriBio, Centre for AgriBioscience, 5 Ring Road, Bundoora, Victoria 3083, Australia; Department of Ecological, Plant and Animal Science, School of Agriculture, Biomedicine and Environment, La Trobe University, 1 Kingsbury Drive, Bundoora, Victoria 3083, Australia

## Abstract

Plants orchestrate tissue-specific metabolic responses to osmotic stress, a major determinant of drought tolerance and crop productivity. Yet how leaf and root responses are coordinated to confer tolerance remains poorly understood. Here we show that drought tolerance in wheat is associated with a reproducible architectural asymmetry between tissue-level metabolomic correlation networks under controlled osmotic stress. In a drought-tolerant genotype, leaf networks are ∼40% denser and highly integrated, consistent with rapid photosynthetic adaptation, whereas root networks are modular and fragmented, consistent with localized responses. Temporal analysis revealed a decline in cross-tissue coordination, from early synchrony (*ρ* ≈ 0.546) toward greater tissue-specific organization (*ρ* ≈ 0.350) under prolonged stress, a pattern absent in the susceptible genotype. Bayesian structure learning provided convergent support for these architectures as statistically robust, nonrandom network organizations (*P* < 0.001). Our findings suggest that drought tolerance is associated with contrasting tissue-level correlation-network organization and temporally structured leaf–root coordination under osmotic stress. This framework advances our understanding of stress adaptation and provides a conceptual basis for breeding climate-resilient crops by targeting key network properties.

## Introduction

Leaves and roots mount coordinated metabolic responses to osmotic stress, yet the architectural principles that organize their tissue-specific networks over time remain unresolved (Munns and Tester 2008; Chen and Jiang 2010; Li et al. 2021). This gap limits mechanistic models of whole-plant homeostasis and is increasingly urgent given the rising frequency and intensity of drought linked to climate change (Obata and Fernie 2012; Chiang et al. 2021).

Network analysis provides a strong framework to address this challenge. In biological systems, integrated, small-world–like architectures support rapid, coherent control, whereas modular organization confers robustness and enables localized adaptation, properties predicted to be differentially advantageous in leaves versus roots under water deficit (Watts and Strogatz 1998; Albert et al. 2000; Barabási and Oltvai 2004; Hodge 2004; Chaves et al. 2008). Although metabolomics has revealed rich, tissue-specific reprogramming, most studies focus on individual metabolites or pathways, leaving the roles of network topology, hub structure, and temporal coordination across tissues largely unexplored (Toubiana et al. 2013; Sweetlove and Fernie 2018). The observation that leaves and roots can respond differentially at the metabolic level further underscores the need to resolve architecture, not merely the magnitude and direction of change (Gargallo-Garriga et al. 2014).

Here, we tested the hypothesis that drought tolerance in wheat (*Triticum aestivum* L.) is associated with asymmetric tissue architectures that reconfigure over time. Using 2 bread-wheat genotypes with consistently contrasting drought-response rankings across prior field and controlled-environment studies, we paired time-resolved LC–MS metabolomics with network analysis to ask whether genotype-dependent tolerance is reflected in leaf–root network organization (Bennani et al. 2017; Yadav et al. 2019; Hone et al. 2021; Shoaib et al. 2025). We found that leaves of the tolerant genotype assembled denser, more integrated networks, whereas roots resolved into more modular structures. Cross-tissue coordination declined as stress persisted, signaling a shift from whole-plant synchrony to tissue-specialized control (Miao et al. 2017; Fait et al. 2020). These architectures are supported by benchmarking against degree-preserving null models and differ from random expectation (Clauset et al. 2009; Langfelder et al. 2011). To assess generality, we applied an identical pipeline to an independent Arabidopsis dataset. This analysis supports the broader leaf–root architectural scaffold, while showing that allocation within this conserved scaffold can be retuned by hormone signaling in an engineered line (35S:BRL3-GFP), shifting integration toward roots (Lozano-Elena et al. 2022). These findings support a conserved architectural asymmetry and temporal decoupling as candidate organizing principles of plant resilience. This framework, in turn, provides network properties—module stability, hub distribution, and cross-tissue coherence—as candidate quantitative markers for breeding climate-resilient crops (Barabási et al. 2011; Riedelsheimer et al. 2012).

## Results

### A reproducible architectural asymmetry distinguishes leaf and root metabolomic correlation networks

**Figure 1:**
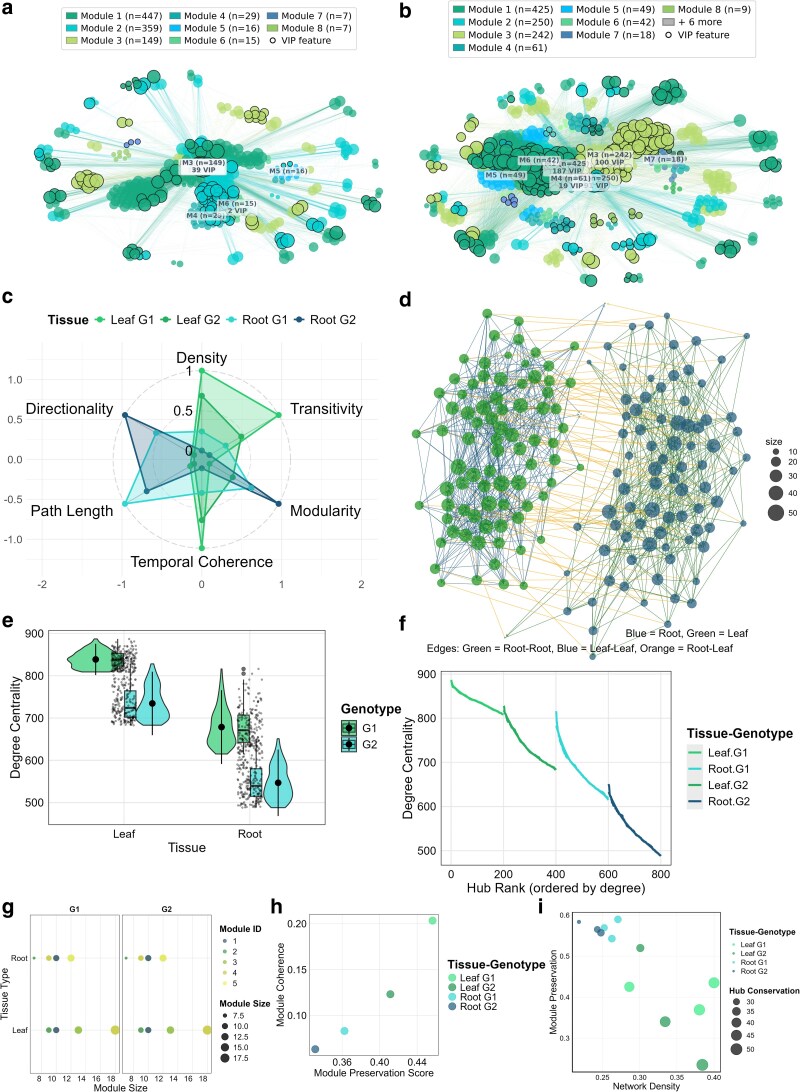
Contrasting leaf–root metabolomic correlation-network architectures and genotype-dependent stability under osmotic stress. Networks were built from high-confidence LC–MS features; edges denote significant Spearman correlations (|*ρ*| ≥ 0.70, FDR < 0.05). Nodes represent molecular features (size ∝ degree). VIP features (VIP > 1.0) are highlighted (black outline). a and b) Leaf (a) and root (b) metabolomic correlation networks. Node color denotes Louvain modules—algorithmically detected communities of co-varying features; colors do not imply predefined biochemical pathway annotation. Labels show module size; node size ∝ degree; black outlines mark VIP features (VIP > 1.0). Leaves are dominated by a few large modules connected through high-degree hubs, whereas roots partition into many smaller modules with fewer cross-module connections. c) Radar plot (*z*-scored metrics) comparing density, transitivity, modularity, path length, directionality (edge-sign asymmetry), and temporal coherence across tissue–genotype networks (leaf/root × G1 tolerant/G2 susceptible). d) Bayesian network inferred from the same features recapitulates the tissue partitioning observed in correlation networks; cross-tissue links are highlighted. e and f) Hub structure. e) Degree-centrality distributions show higher and more concentrated hub degrees in leaves; (f) rank–degree decay is steeper in leaves and shallower in roots. g) Module spectrum by tissue and genotype indicates distributed, mid-sized modules in roots versus fewer, larger modules in leaves. h) Module preservation versus coherence from bootstrap/permutation analysis (1,000 resamples) shows higher stability for G1. I, Network-level trade-off between density and preservation; point size encodes hub conservation through time. Roots prioritize preservation; leaves balance connectivity and preservation. (See Materials and Methods for metric definitions and resampling procedures.)

A total of 2,471 molecular features were profiled and tissue- and genotype-specific metabolomic correlation networks were constructed; primary analyses focused on 964 high-confidence features (VIP > 1.0). Edges represented significant metabolite co-abundance associations based on Spearman correlations (|*ρ*| ≥ 0.70, Benjamini–Hochberg FDR < 0.05; Materials and Methods), rather than biochemical reactions, fluxes, or causal regulatory interactions. In the drought-tolerant genotype (G1), leaf networks had higher edge density (fraction of possible edges present) than roots (0.354 vs. 0.192; empirical tests against degree-preserving edge-swap nulls with FDR control unless otherwise noted), higher transitivity (global clustering coefficient; 0.740 to 0.804 vs. 0.686 to 0.714), and shorter mean path length (mean shortest-path length on the largest connected component; 2.04 to 2.17 vs. 2.29 to 2.44). These patterns were consistent across time points and biological replicates (Figs. 1c and S9d to f).

Root networks in G1 were more fragmented and modular than leaf networks, with higher modularity *Q* (stronger separation into communities relative to degree-matched null expectation; *Q* = 0.213 to 0.288 vs. 0.097 to 0.162) and approximately 3-fold more disconnected components (18 to 21 vs. 6), indicating greater fragmentation of correlation structure (Fig. 1a to c). In a singleton audit at |*ρ*| ≥ 0.70 using the combined VIP > 1.0 feature set, singleton nodes (degree 0) were consistently observed (39 to 78 per network across tissue–genotype combinations). These singleton features showed no elevation in preimputation missingness relative to connected nodes (median 0.0% for both groups), and no features were excluded due to complete absence of measurements (all-NA dropped = 0). The largest connected component comprised 83.1% to 89.6% of nodes (Table S8). Hub organization also differed: connectivity in G1 leaves was concentrated in a smaller set of central hubs, whereas in root it was more broadly distributed (Fig. 1e and f). Across genotypes, the susceptible line (G2) received identical osmotic treatment yet exhibited only attenuated separation of these metrics (Fig. 1c, e, and f), indicating that the pronounced leaf–root asymmetry is associated with the tolerant genotype rather than being a uniform consequence of osmotic stress exposure.

We next assessed network preservation and within-module coherence (Langfelder et al. 2011). Module preservation and coherence were higher in G1 than G2 in both tissues: leaf preservation/coherence was 0.375/0.600 and root preservation/coherence was 0.357/0.497, compared with 0.354/0.482 in G2 leaves and 0.328/0.451 in G2 roots (Fig. 1h and i). A complementary Bayesian network analysis corroborated the tissue-specific organization (Figs. 1d and S10a to c; Table S10). Together, the metrics indicate a reproducible architectural asymmetry between leaves and roots, strongest in G1.

### Temporal dynamics reveal cross-tissue decoupling and tissue-specific responses in the tolerant genotype

**Figure 2:**
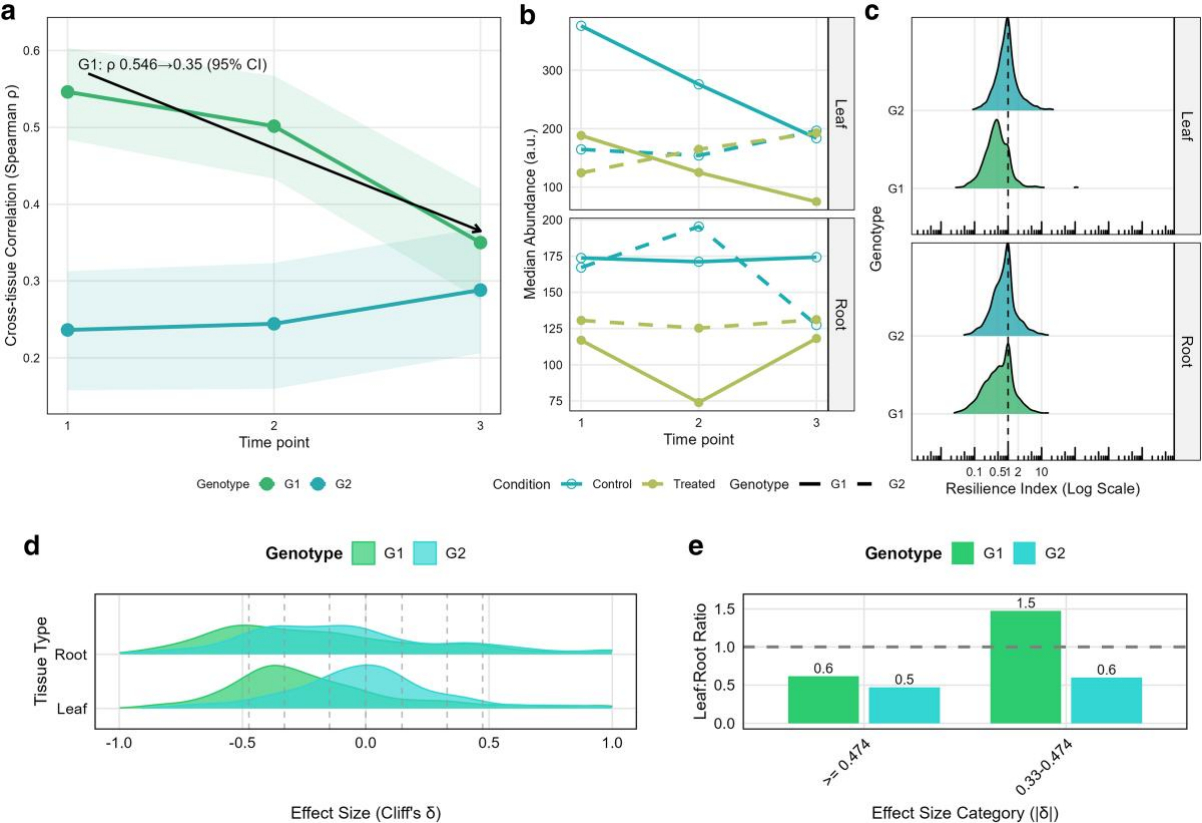
Coordinated decoupling and asymmetric tissue-level responses underpin drought tolerance in wheat. a) Cross-tissue coordination (leaf–root Spearman *ρ* between matched RI vectors on a fixed shared metabolite set, *n* = 668; bootstrap 95% CI from 5,000 resamples) declines progressively in G1, from early synchrony to later tissue-specialized control, while G2 remains lower and comparatively stable. b) Tissue-specific trajectories of median metabolite abundance show divergent responses across genotypes, treatment conditions, tissues, and time points. c) Distributions of the RI (median treated/control abundance; log scale) show greater suppression of metabolic stability in G1 tissues compared with G2. d) Effect-size distributions (Cliff’s δ) highlight tissue and genotype asymmetry, revealing a broader effect-size range in the tolerant G1 genotype, with larger magnitudes concentrated in G1 leaves. e) The leaf:root response ratio, binned by effect-size category (|δ|), shows leaf-dominance in G1 for moderate effects (0.33 to 0.474) but root-dominance in the strongest bin (≥0.474); G2 is root-dominant across bins (ratios < 1).

**Figure 3:**
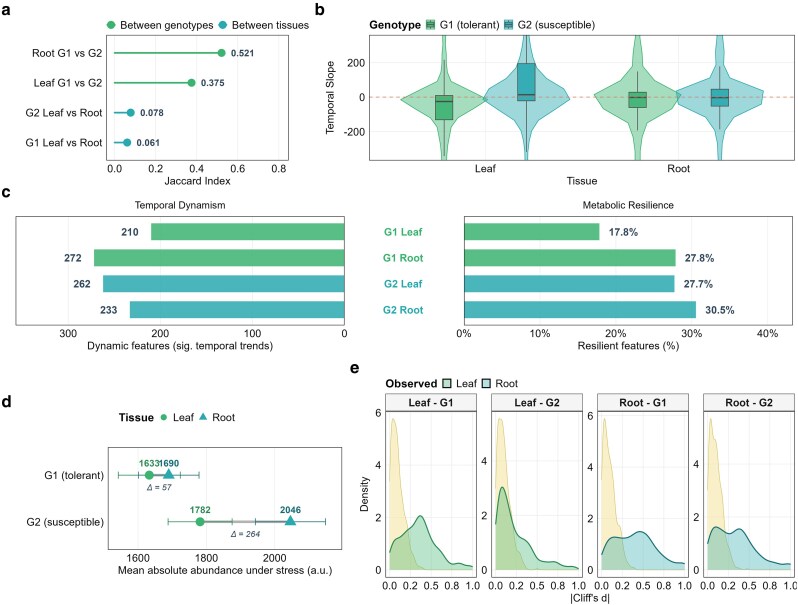
Network organization and feature dynamics support the robustness of tissue-specific adaptation. a) Hub-set overlap, calculated from the top-200 ranked hub lists, shows limited leaf–root overlap within each genotype (G1: 0.061; G2: 0.078) but higher between-genotype similarity within tissues (leaf: 0.375; root: 0.521), indicating that hub identity is primarily tissue-determined. Core top-20 hub-overlap sensitivity across thresholds is reported separately in Table S5. b) Distributions of temporal slopes for VIP metabolites in stressed samples reveal broad, heterogeneous dynamics across tissues and genotypes. c) Butterfly summary contrasting temporal dynamism (number of features with significant temporal trends; left) and metabolic resilience (fraction of features stable at the final time point relative to control; right) for each tissue–genotype combination. In G1, roots exceed leaves for both dynamism and resilience, whereas in G2 dynamism is reversed (leaf > root) and resilience differences are smaller. d) Mean absolute metabolite abundance in stressed samples (95% CI) per tissue and genotype; the root–leaf difference is modest in G1 (D = 57 a.u.) but larger in G2 (D = 264 a.u.). e) Observed |Cliff's δ| distributions (stress vs. control) compared with a permuted-label null, faceted by tissue–genotype; observed distributions are shifted toward larger effect sizes, supporting nonrandom metabolic responses across conditions.

Time-resolved network behavior under osmotic stress was next examined. Cross-tissue coordination was quantified as the Spearman correlation between matched leaf and root Resilience Index (RI) vectors using a fixed shared metabolite set across genotypes and time points (*n* = 668 metabolites). In G1, cross-tissue coordination declined from the initial to later stress stages (*ρ* = 0.546, 95% confidence interval [CI] 0.484 to 0.603, to *ρ* = 0.350, 95% CI 0.275 to 0.420; Fig. 2a), consistent with a transition from early leaf–root synchrony toward greater tissue-specific organization. This tissue-specific organization was further supported by limited leaf–root overlap among the ranked hub lists shown in Fig. 3a (Jaccard = 0.061 in G1 and 0.078 in G2), with consistently low overlap among core top-20 hubs observed across threshold combinations (Table S5). In G2, cross-tissue correlation remained lower and relatively stable (*ρ* = 0.236 to 0.288) over the same interval.

Median metabolite abundance trajectories revealed divergent responses across tissues, genotypes, treatment conditions, and time points (Fig. 2b). Consistent with this, variable importance in projection (VIP) metabolites showed broad, dynamic temporal responses in G1 (Fig. 3b). In G1, roots had more metabolites with significant temporal trends than leaves (272 vs. 210), whereas in G2 leaves exceeded roots (262 vs. 233) (Fig. 3c). Mean absolute metabolite abundance under stress was higher in roots than in leaves, with a larger tissue difference in G2 (Fig. 3d). Observed effect sizes exceeded permutation-derived null distributions across categories (Fig. 3e).

The overall suppression of metabolic stability was greater in G1 tissues (Fig. 2c). Consistent with this asymmetry, the fraction of temporally stable (“resilient”) features at the final time point was higher in G1 roots than in leaves (27.8% vs. 17.8%), whereas G2 roots and leaves were more similar (30.5% vs. 27.7%) (Fig. 3c). Distributional analyses of effect sizes (Cliff’s δ, |δ|) further resolved tissue differences (Fig. 2d): in G1, leaf:root ratios exceeded 1 for moderate effects (0.33 to 0.474) but fell below 1 in the strongest effect-size category (≥0.474), indicating a nuanced allocation of response across tissues (Fig. 2e).

Collectively, the time-series analyses document (i) a decline in cross-tissue coordination in G1 that was not observed in G2; (ii) distinct within-tissue trajectories, with G1 roots exhibiting more dynamic temporal reprogramming than leaves; and (iii) a tissue-biased allocation of metabolic resilience in G1, where roots maintain a large stable core, a specialization absent in G2.

### Observed network architectures are robust and nonrandom

For tissue-level comparisons which form the primary basis for the asymmetry conclusions, median per-feature statistical power was 0.639, with 38.3% of comparisons exceeding the 0.80 threshold (Table S6; Fig. S9a to c). A multi-tiered validation framework was implemented to assess robustness and departure from random structure. First, at the module level, we compared a composite integration score against a module preservation score. In G1, the leaf network attained the highest integration score (0.600) together with a high preservation score (∼0.375); the root network was also highly preserved (∼0.36) (Fig. 1h). Corresponding scores were lower in G2 (leaf preservation/coherence 0.354/0.482; root 0.328/0.451) (Fig. 1h). Differences were assessed by permutation within genotype/tissue strata with BH FDR correction (Materials and Methods).

**Figure 4:**
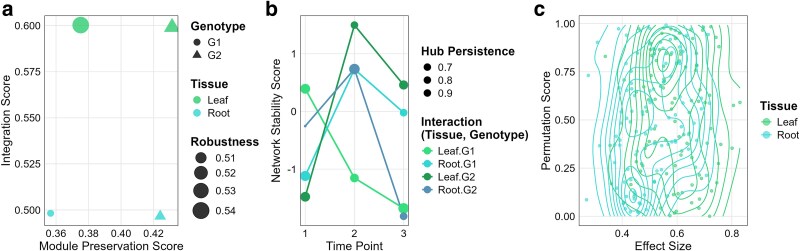
Multi-level validation demonstrates robustness and departure from random structure. a) Module-level validation relating composite integration (*y*-axis) to module preservation (*x*-axis). Points represent leaf and root modules; shape denotes genotype; point size encodes robustness. Leaf modules, especially in G1, occupy the high-integration, high-robustness region; roots favor preservation at lower integration. b) Network stability trajectories across time for each tissue–genotype combination; point size encodes hub persistence (fraction of top hubs retained). Stability dynamics mirror decoupling, with the strongest mid-experiment divergence and partial convergence thereafter. c) Relationship between effect-size values and permutation-derived scores for leaf and root validation outputs. Points represent individual validation values; contours show the two-dimensional kernel density of the plotted points.

Second, we evaluated dynamic stability across the stress period using a network stability score. In G1 leaves, stability changed from ∼0.3 at the initial time point to −1.7 at the final time point; G2 networks showed larger oscillations without a consistent trajectory (Fig. 4b). Hub persistence contributed to the stability summary (point size in Fig. 4b; Materials and Methods). Temporal differences in stability were tested by permutation across time labels with FDR control (Materials and Methods).

Third, we benchmarked the observed networks against explicit nulls using 5,000 degree-preserving permutations (edge-swapping null model) (Clauset et al. 2009). For each network, we compared observed topology-sensitive metrics—modularity Q, transitivity, and average clustering—against the null distributions generated from the 5,000 degree-preserving permutations. These metrics deviated strongly from null expectations (BH-adjusted P < 0.001 across tested summaries; Methods). In addition, effect-size distributions differed between tissues: leaf values showed a higher mean effect size (0.547) and tighter clustering than root values (mean 0.396), with tissue-specific clustering in effect-size–permutation-score space (Fig. 4c). Results were stable across alternative correlation thresholds (Fig. S8a to c), VIP cut-offs (Fig. S8d and e), and permutation seeds (Materials and Methods); across all 20 paired comparisons (2 VIP thresholds × 5 |*ρ*| thresholds × 2 genotypes), the leaf-denser/root-more-modular asymmetry was invariant (Table S4). Network-level metric estimates were stable under resampling (Fig. S9d to f). Network metrics showed low sensitivity to replicate composition (jackknife CV < 10% for all metrics; density and modularity range 0.060 to 0.099, transitivity range 0.008 to 0.026; Table S7), and cluster-bootstrap resampling (200 iterations) preserved the leaf-denser/root-more-modular ordering; for G2, the modularity difference (leaf–root) was Δ = −0.089 (95% CI: −0.195, −0.004).

Finally, convergent evidence was sought from Bayesian network structure learning, which infers conditional dependencies under a directed acyclic graph (DAG) constraint. For G1 leaves, the observed number of edges (493) exceeded the expected count under the permuted-data null (106); G1 roots showed a similar excess (406 observed vs. 106 expected) (*P* < 0.001 for both) (Figs. 1d and S10a to c; Table S10). These analyses converge on the same conclusion under distinct modeling assumptions: the tissue-specific networks are reproducible across time, preserved within modules, and statistically distinct from random structure generated under the Bayesian permuted-data null. Repeating structure learning under maxp = 5 and maxp = 3 preserved the leaf–root ordering and retained 88.6% to 92.7% of constrained arcs within the unconstrained scaffold (Fig. S10a to c; Table S10).

### Cross-species comparison

**Figure 5:**
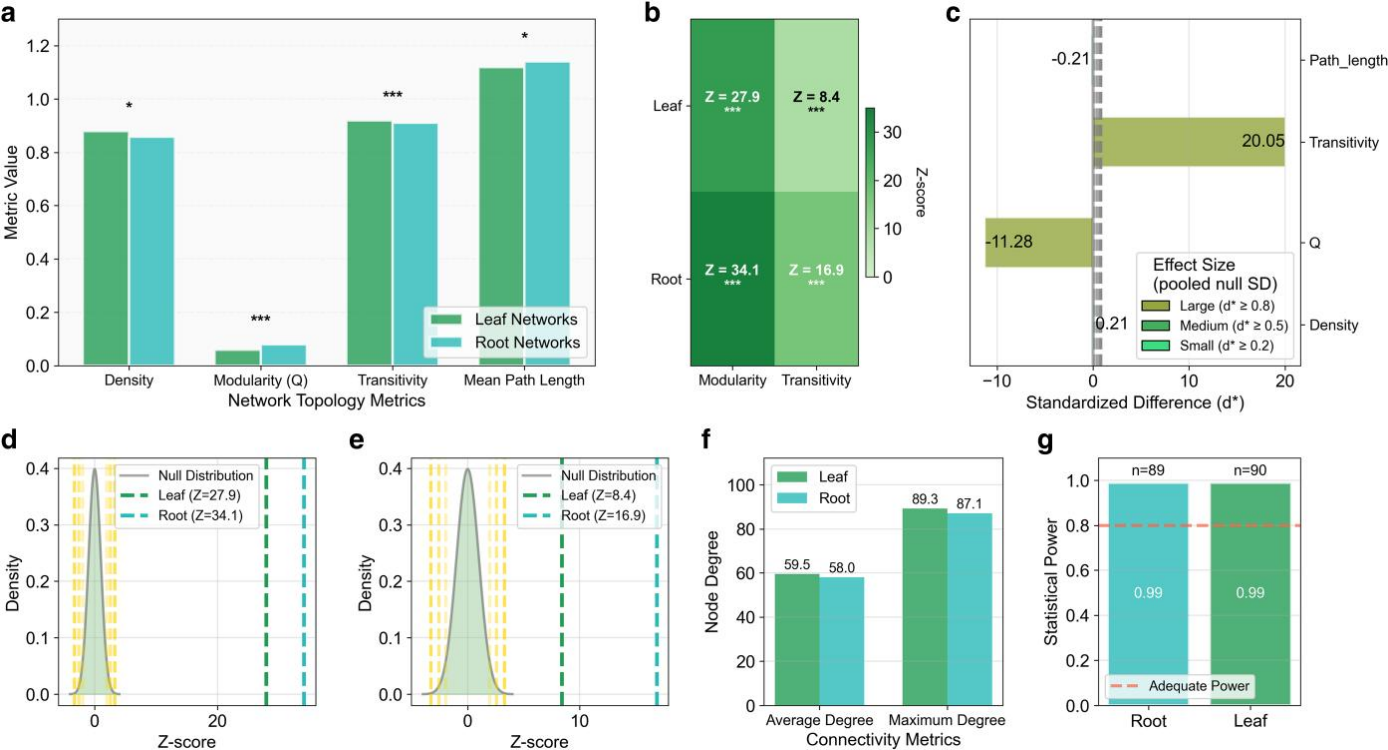
Cross-species comparison in *A. thaliana* provides supporting evidence for a similar tissue-level scaffold. An identical analytical pipeline was applied to an independent Arabidopsis dataset (MTBLS2289; 62 shared metabolites) to test the generalizability of the wheat network architecture. a) A similar tissue-level scaffold was observed: shoot networks (*n* = 90) are denser and more efficient, whereas root networks (*n* = 89) are more modular. Asterisks denote effect-size magnitude (|*d*| ≥ 0.2, **≥0.5, ***≥0.8; d* computed using pooled-null SD). b) A *Z*-score heatmap shows exceptionally large deviations from degree-preserving nulls for modularity and transitivity in both tissues (*P* < 0.001). c) Standardized differences (d*, pooled-null SD) quantify the shoot–root differences for each core metric. d and e) Null-distribution overlays visualize the departures for modularity and transitivity. f) Connectivity metrics indicate slightly higher average and maximum degree in shoots, consistent with the integrated-shoot motif. g) Sample sizes and the resulting statistical power (0.99 in both tissues) are shown for context. Methodology: Spearman |*ρ*| ≥ 0.30; FDR < 0.05; 200 degree-preserving nulls.

To test generality across species, we applied the identical pipeline to an independent public dataset in Arabidopsis (MTBLS2289; shoot *n* = 90, root *n* = 89; 62 shared metabolites, 6.4% of the 964 wheat high-confidence features—a deliberately conservative cross-species test of scaffold detectability). In MTBLS2289, the aerial compartment corresponds to shoot (rosette) tissue harvested from 3-wk-old prebolting plants, and we refer to this compartment as shoot (rosette) throughout (Lozano-Elena et al. 2022). This cross-species comparison provides supporting evidence for a similar tissue-level scaffold under reduced metabolite coverage: shoot networks were denser and more efficient, whereas root networks were more modular (shoot vs. root: density 0.880 vs. 0.859; modularity *Q* 0.060 vs. 0.080; mean path length 1.120 vs. 1.141; transitivity 0.920 vs. 0.911) (Fig. 5a; effect sizes in Fig. 5c). The standardized differences for modularity and transitivity were particularly large (d* = −11.28 and 20.05, respectively), while the effects for density (d* = 0.21) and path length (d* = −0.21) were small, supporting nontrivial architectural contrasts (Fig. 5c). Furthermore, degree-preserving nulls had very large deviations from the observed data (eg *Z*[*Q*] 27.9 shoot, 34.1 root), confirming that these architectures are highly nonrandom and statistically robust (Fig. 5b, d, and e).

Consistent with the integrated-shoot motif, shoot nodes are slightly more connected than root nodes (average degree 59.5 vs. 58.0; maximum degree 89.3 vs. 87.1) (Fig. 5f).

## Discussion

### A conserved architectural scaffold as an organizing principle

Our cross-species analysis points to a recurring organizing principle of plant resilience: a tissue-specific scaffold where leaves preferentially assemble dense, integrated networks and roots resolve into more modular architectures. That a similar scaffold was observed in an independent Arabidopsis dataset supports the interpretation that this tissue-level organization may represent a broader organizational pattern for balancing rapid coordination against local robustness. This modularity is a hallmark of biological systems, from metabolic pathways to protein interaction networks (Ravasz et al. 2002; Kitano 2004; Newman 2006). The large deviations of these architectures from degree-preserving null models, together with the scale-free organization reported for metabolic networks and the nonrandom topology of biological interaction networks, indicate that they are not stochastic by-products but a defining feature of plant network organization (Jeong et al. 2000; Maslov and Sneppen 2002).

### Scaffold tunability: hormone signaling modulates network allocation

Strikingly, although the scaffold is conserved, allocation within it is tunable rather than fixed. The drought-tolerant wheat genotype concentrates integration in leaves, whereas the engineered Arabidopsis 35S:BRL3-GFP line reallocates integration toward roots in our cross-species analysis, reported by Lozano-Elena et al. (2022). This contrast does not imply independent evolutionary “solutions”; in Arabidopsis, it reflects constitutive overexpression of the vascular brassinosteroid receptor BRL3, representing a targeted perturbation of brassinosteroid signaling (Fàbregas et al. 2018). Brassinosteroid signaling has established cell-type-specific roles in root development and adaptation to abiotic stress, supporting the interpretation that network allocation is a regulatable property that can be retuned by signaling inputs (Planas-Riverola et al. 2019). More broadly, natural genetic variation and regulatory plasticity are both recognized routes by which plants shift stress-responsive phenotypes under changing environments (Des Marais et al. 2013), and root-system plasticity is itself a central component of abiotic-stress adaptation (Karlova et al. 2021). That wheat genotypes and 35S:BRL3-GFP represent different deployments of a shared architectural template is a conclusion that follows directly from the network analyses presented here.

### Functional rationale for tissue-specific network design

This architectural dichotomy maps onto organ function. The leaf-centric integration in tolerant wheat, characterized by short path lengths and high transitivity, is well suited to coordinated control of photosynthesis and carbon export (Flexas et al. 2012). This “small-world” property is consistent with efficient information transfer across the network (Watts and Strogatz 1998; Latora and Marchiori 2001). Conversely, root modularity is consistent with a robust strategy for navigating a patchy soil environment, enabling semi-autonomous modules to manage local osmotic adjustment and nutrient uptake without destabilizing the entire plant (Hodge 2004; Giehl and von Wirén 2014). The root-centric integration in the engineered Arabidopsis line (35S:BRL3-GFP) is likewise consistent with a strategy that prioritizes stabilization of water and ion balance at the soil interface (Lozano-Elena et al. 2022). It should be emphasized that these functional interpretations are grounded in prior network theory and physiology, not inferred directly from correlation structure. Co-abundance networks capture co-variation under shared conditions but do not establish flux, regulation, or causal direction. Causal links will require orthogonal validation (eg stable-isotope fluxomics, targeted perturbations, and time-resolved multi-omic integration), as outlined in the Limitations section.

### Temporal decoupling as dynamic control

Beyond spatial organization, the system exhibits a distinct temporal architecture. The observed decline in cross-tissue coordination in wheat represents a shift from an initial, synchronized “whole-plant alarm” to a later phase of tissue-specialized control. This coordination relies on rapid, long-distance signaling molecules and ions that communicate stress from root to shoot (Choi et al. 2014; Zandalinas et al. 2020; Li et al. 2021). This dynamic, also seen in other species (Miao et al. 2017; Fait et al. 2020), helps avoid a “rigidity trap” (Scheffer et al. 2009). Systemic signals such as ABA initially synchronize the response (Cutler et al. 2010), then cede control to allow leaves and roots to optimize distinct tasks (Tardieu et al. 2018).

### Robustness and sensitivity of findings

The core conclusions do not hinge on a single analytic choice. We confirmed this by (i) applying an identical, seeded pipeline across species (Zhang and Horvath 2005; Langfelder and Horvath 2008); (ii) showing that the scaffold’s qualitative features were invariant to reasonable changes in correlation cut-offs (Fig. S8a to c) and VIP feature-selection thresholds (Fig. S8d and e;  Rubinov and Sporns 2010; Van Wijk et al. 2010); (iii) benchmarking against rigorous, degree-preserving nulls (Maslov and Sneppen 2002); and (iv) obtaining convergent evidence from Bayesian structure learning (Friedman et al. 2000; Scutari 2010). Module-preservation analyses further indicate the coarse scaffold is stable even as specific network edges re-wire under stress (Langfelder and Horvath 2008; Langfelder et al. 2011).

### Implications for phenotyping and breeding

This framework provides candidate quantitative traits for crop improvement. Network properties such as module stability, hub distribution, and cross-tissue coherence (eg leaf–root eigengene coupling) can be summarized into features that are amenable to association with genetic variation and to use as covariates or predictors in genomic prediction pipelines (Zhang and Horvath 2005; Goddard and Hayes 2009; Riedelsheimer et al. 2012; de los Campos et al. 2013; Crossa et al. 2017; Hickey et al. 2019). In practice, network-derived features can complement genomic and metabolomic markers to improve predictive accuracy and interpretability under stress, enabling selection for genotypes that achieve a favorable balance of leaf integration and root modularity. Because the scaffold is conserved, the key breeding lever is allocation—how each genotype positions integration within the shared architecture; establishing the stability and predictive value of these network-derived traits under field drought is an important next step.

### Limitations and future work

All experiments were performed in controlled hydroponics to isolate osmotic stress from edaphic, biotic, and climatic variability inherent to field drought. This design enabled tightly synchronized stress onset and rapid, soil-free root harvesting, which are prerequisites for time-resolved, tissue-resolved network reconstruction. The 2 wheat genotypes were chosen for drought-response rankings that are reproducible across environments and controlled-environment metabolomic responses have been shown to predict field drought performance in wheat (Yadav et al. 2019). Nonetheless, establishing the stability and predictive value of the network-level architectural features reported here under field drought, including heterogeneous soil moisture and rhizosphere interactions, remains an important next step toward breeding utility. Because the present controlled-environment design isolates a single osmotic stressor, determining whether this architecture is shared with other abiotic or biotic stresses will require comparison against heat, salinity, and pathogen challenge under similarly controlled designs—a natural extension of the present work.

The profound differences between wheat genotypes and Arabidopsis signaling mutants naturally constrain direct mechanistic mapping. Essential next steps include: (i) using stable-isotope fluxomics (^13^C-MFA) to ground inferred coordination in measured metabolic flux (Ma et al. 2014); (ii) deploying tissue-specific CRISPR/Cas9 perturbations of predicted hub genes to functionally test their roles as network control points (Bortesi and Fischer 2015); and (iii) integrating multi-omic layers to build causal models of how regulation gives rise to network architecture (Hasin et al. 2017). Because these are co-abundance networks, topology metrics quantify statistical organization of metabolite covariation rather than causal regulation or biochemical flux; establishing causal links to physiology will require orthogonal approaches such as stable-isotope flux measurements, targeted tissue-specific perturbation experiments, or time-resolved multi-omic integration.

The primary wheat analysis is based on 2 contrasting genotypes selected for mechanistic dissection under a tightly synchronized, time-resolved sampling design. Controlled-environment metabolomic responses have been shown to predict field drought performance in wheat (Yadav et al. 2019). This focused design enabled high-resolution, time-resolved tissue sampling that the network analyses require, whereas extending the protocol to larger genotype panels without scaling personnel would risk timing artifacts that bias network metrics. Focused contrasting-genotype designs of this type are standard in network biology when the aim is mechanistic dissection rather than population-level inference (Toubiana et al. 2013; Miao et al. 2017). Nevertheless, the generality of these architectural properties across broader wheat diversity remains to be established, and extension to diversity panels or recombinant populations is a logical next step for testing transferability, genetic basis, and breeding relevance (Riedelsheimer et al. 2012; Hill et al. 2013).

In sum, drought tolerance operates on a conserved leaf–root architectural scaffold, a shared organizational template in which leaves favor rapid, coordinated integration, whereas roots favor modular, locally robust responses. What is tunable is not the scaffold itself, but the allocation within it: the drought-tolerant wheat genotype concentrates integration in leaves, whereas engineered modulation of brassinosteroid signaling in Arabidopsis reallocates integration toward roots, demonstrating that hormone signaling can retune network allocation within a conserved framework. Recognizing that plants deploy, rather than reinvent, this shared architecture reconciles cross-species variation and reframes resilience as an emergent property of how that scaffold is organized across tissues and time. This perspective highlights network-derived features, including module stability, hub distribution, and cross-tissue coherence, as candidate quantitative traits for breeding climate-resilient crops.

## Materials and methods

### Plant growth conditions and stress treatments

We used 2 bread wheat genotypes with reproducible, contrasting drought responses—G1 (Gladius; drought-tolerant) and G2 (DAS5_003811; drought-susceptible)—selected on the basis of consistent evidence across independent field and controlled-environment studies (Bennani et al. 2017; Yadav et al. 2019; Hone et al. 2021; Shoaib et al. 2025). This phenotypic anchor enabled focused mechanistic analysis of tissue-specific network architecture. All treatments employed 7 biological replicates per genotype per time point to ensure adequate power for time-series and network statistics (Fig. S9a to c). Each biological replicate was an independent, individually grown plant; leaf and root tissues were harvested and extracted separately from each plant, with no pooling of material across plants.

Plants were cultivated in an aerated hydroponic system under a 22 h light/2 h dark cycle (22 °C during light; 17 °C during dark) with 60% to 70% humidity (Ghosh et al. 2018). The pH was maintained at 6.5 through daily monitoring, and the nutrient solution (Table S2) was replaced every 3 d during the first 4 wk and daily thereafter. Initially, plants of each genotype were grown in a shared reservoir; 24 h before treatment they were transferred to individual buckets to ensure accurate application. Hydroponic culture was used to enable tightly synchronized stress onset and rapid, low-contamination root harvest, which is difficult to achieve at comparable temporal resolution in soil-grown plants. Both genotypes were sown concurrently and grown in parallel under identical controlled-environment conditions; stress was initiated concurrently for both lines. Sampling time is indexed by days after sowing and days after stress onset.

Two from the following Two osmotic stress protocols were applied (*n* = 7 biological replicates each) to capture variation in drought timing and severity: Batch 1 received acute stress (0.3 M sorbitol, Days 38 to 41) whereas Batch 2 underwent prolonged stress (0.15 M sorbitol, Days 37 to 46). Both followed identical pretreatment protocols, with time Points 1, 2, and 3 corresponding to Days 1, 2, and 3 for Batch 1, and to Days 3, 6, and 9 for Batch 2, respectively. This dual-protocol approach reduced dependence on a single sorbitol concentration or treatment duration, while preserving the primary experimental focus on controlled osmotic stress.

### Sample preparation

Root and leaf tissue were harvested (leaf material from the youngest fully expanded leaf at each harvest), quenched in liquid nitrogen, and stored at −80 °C until further analysis. Samples were freeze-dried using a Christ Alpha 1-4 LDplus (Martin Christ, Germany), with roots in perforated Eppendorf tubes and leaves arranged on racks in opened Ziplock bags. Freeze-dried samples were then ground using a Geno/Grinder 2010 (Cole-Parmer, Illinois, USA) at 1,200 rpm for 4 min, with 30-s breaks each minute. For untargeted analysis, 4 ceramic balls (3.5 to 4.1 mm) were used per sample, whereas targeted analysis employed 6 balls (two 4.1 to 4.8 mm and four 2.8 to 3.2 mm). After grinding, 20 mg of material was extracted with 1 mL of 80% methanol, shaken for 5 min, sonicated for 10 min, shaken again for 10 min, and incubated at room temperature for 1 h. The extracts were then centrifuged at 12,300 rpm for 10 min, and 100 *µ*L of the supernatant was aliquoted into HPLC vials (Roessner et al. 2001; Rochfort et al. 2008).

### LC–MS analysis

Metabolite separation and analysis were performed using a Vanquish UHPLC system coupled with a Q Exactive Plus Orbitrap mass spectrometer (Thermo Scientific). Chromatographic separation was performed on a C18 column (2.1 × 100 mm, 1.7 *µ*m) at 30 °C, using water (A) and acetonitrile (B), each containing 0.1% formic acid, as mobile phases (Rochfort et al. 2008). The gradient (0.3 mL/min) progressed from 2% to 100% B over 11 min, held for 4 min, then returned to initial conditions (5-min re-equilibration). Mass spectrometric detection employed heated electrospray ionization in positive and negative modes (spray voltages: 3,600 and 3,300 V; capillary temperature: 300 °C; sheath gas: 28 arbitrary units; S-lens RF: 64). Full MS scans (m/z 100 to 1,500) were acquired at 70,000 FWHM resolution (m/z 200), with an injection volume of 5 *μ*L. Internal standards were included in each sample to monitor extraction/instrument stability and support within-run normalization. Pooled QC samples were regularly inserted to assess technical variability and ensure consistent performance across the run (Gika et al. 2007; Dunn et al. 2011).

### LC–MS data processing and quality control

#### Initial data processing

Raw LC–MS data were processed using Genedata Expressionist Refiner MS 18.0.1. The workflow comprised data import, chromatogram extraction, chemical noise subtraction (via a moving average algorithm), retention time alignment (using a pairwise alignment-based tree method), peak detection (employing a curvature-based algorithm with subsequent refinement and consistency filtering), and isotope clustering (using a peptide isotope shaping method). Separate mode-specific workflows were applied for positive and negative ionization. Detailed parameters for each step are provided in Table S3.

#### Data quality control and feature selection

Data preprocessing followed a rigorous multi-step workflow to ensure data quality and reliability (Fig. S1). Initial data filtering excluded columns with fewer than 3 replicates, reducing molecular features from 4,255 to 1,789 in negative mode and from 3,199 to 1,350 in positive mode. Despite this reduction, missing values persisted, a common issue in LC–MS studies (Karpievitch et al. 2012; Kokla et al. 2019). Missingness accounted for 12.67% of values pooled across all samples and ionization modes (leaf: 17.21% to 17.62%; root: 7.80% to 9.55%) and was imputed using Random Forest; missingness mechanism assessment and imputation method selection are described in Fig. S2a to f. Outlier detection employed an Isolation Forest algorithm, followed by asinh transformation, which reduced data variability (CV from 0.876 to 0.206). The final dataset comprised 2,471 molecular features (1,398 from negative mode, 1,073 from positive mode), with tissue-specific distributions detailed in Supplementary material.

### Network analysis and statistical methods

#### Initial statistical analysis

The analytical framework combined feature-level statistics and network-based analyses to address distinct biological questions (Fig. S7). Feature-level tests and VIP prioritization identified metabolites whose abundance changed under stress, whereas metabolomic correlation-network analysis summarized how these features were organized into hubs, modules, and tissue-level co-abundance structures. Spearman correlations identified co-abundance associations, network topology quantified tissue-level organization, and Bayesian structure learning provided a complementary conditional-dependence representation under directed-acyclic-graph assumptions. This layered strategy reduced single-method bias while providing convergent evidence from independent modeling assumptions.

Statistical analysis comprised 6 complementary approaches: (i) tissue comparison (Mann–Whitney *U* tests), (ii) genotype comparison (Cliff's Delta effect sizes), (iii) treatment effects (Wilcoxon/Mann–Whitney tests), (iv) temporal dynamics (Friedman tests), (v) integrated responses (rank-based analysis), and (vi) metabolic resilience (RI = median(treated)/median(control), computed per metabolite within each tissue–genotype–time stratum; bootstrap 95% CI, *n* = 5,000 resamples). RI is a ratio-based operationalization of resilience and the median was used because LC–MS abundances are typically right-skewed and median-based ratios are less sensitive to skew and outliers (Hampel et al. 1986; Lötsch et al. 2024). Multiple testing was controlled using Benjamini–Hochberg FDR (*α* = 0.05). Relationship stability was assessed through bootstrap correlation analysis (5,000 iterations) with 95% CIs. Effect sizes were calculated using Cliff's δ because only 36.27% of feature-level comparisons met Shapiro–Wilk normality in both stressed and control groups after asinh transformation (leaf: 25.31%; root: 45.30%). Statistical power was evaluated through nonparametric simulations (5,000 iterations, *α* = 0.05; Table S6). As a robustness check, for features satisfying normality in both groups, ANOVA and Mann–Whitney *U* were both BH-FDR corrected over the identical normal-only feature subset and concordance of significance calls was assessed (Table S9).

##### Response-Magnitude Index

Directional metabolic change was quantified using the RMI, defined as the signed median Cliff's δ within each tissue × genotype × time point stratum. For each metabolite, Cliff's δ (range −1 to +1) was computed from treated versus control ranks at the same time point; RMI is the median of per-metabolite δ values, preserving direction (positive = higher under stress; negative = lower) while providing a robust summary. We report |RMI| as response intensity when direction is not of interest. RMI summarises distributional shifts independently of absolute concentrations, complementing the median abundance trajectories (Fig. 2b) and the leaf:root ratios of effect-size categories (Fig. 2e). Permutation-based nulls (5,000 iterations; Materials and Methods, Permutation testing) were used to benchmark observed effect sizes. Temporal trends were called significant at FDR < 0.05 (Benjamini–Hochberg).

For genotype-specific analyses, leaf and root tissues were evaluated separately. Temporal patterns were assessed using Friedman tests, with Kendall's *W* employed for effect size estimation. Multiple testing was controlled using both Benjamini–Hochberg FDR and Bonferroni FWER methods (*α* = 0.05), with significance defined primarily by FDR-adjusted *P* < 0.05. Tissue-specific responses were evaluated through Mann–Whitney *U* tests, with *P*-values adjusted within each tissue group to control for multiple comparisons. Tissue structure was further characterized using coefficients of variation and network modularity.

#### Feature selection

Feature selection used Partial Least Squares Discriminant Analysis (PLS-DA) with nested cross-validation (outer: 10-fold, inner: 5-fold) and VIP scoring (threshold >1.0), implemented via custom ScalerPLSPipeline in Python 3.10. First, PLS-DA was performed separately for leaf and root tissues using a nested cross-validation framework (outer CV: 10-fold; inner CV: 5-fold) with stratified sampling. Model optimization employed grid search across scaling methods (Standard, MinMax, Robust Covariance Estimation [Robust]) and components (1 to 10), using mean squared error as the optimization metric. A custom pipeline incorporating ScalerPLSPipeline was implemented to ensure consistent preprocessing across validation folds. Features were ranked using VIP scores, with scores >1.0 considered significant, following the established threshold criterion in metabolomics study (Chong and Jun 2005) (VIP-threshold sensitivity: Fig. S8d and e). To assess sensitivity to this threshold, all analyses were repeated at VIP ≥ 0.8, which retained 1,126 leaf and 1,200 root features compared with 449 and 598 at VIP ≥ 1.0; core architectural conclusions were qualitatively unchanged (Table S4; Fig. S8d and e).

Feature significance was further validated through nonparametric Mann–Whitney *U* tests comparing metabolite distributions between stressed and control conditions along with detailed metabolite characterization (see Supplementary material “Metabolite Analysis Results” section for comprehensive temporal and pathway-level analyses). Multiple testing correction employed both Bonferroni correction for family-wise error rate control and Benjamini–Hochberg procedure for FDR control (*α* = 0.05). The final feature set comprised molecular features that exceeded the VIP threshold and passed both multiple testing corrections. These validated features were annotated with tissue-specific identifiers and compiled into a unified dataset for subsequent network analyses.

#### Network construction and topology analysis

Network architecture was characterized through key topological metrics including network density, transitivity, modularity, component analysis, and mean path length calculations (see Table S1 for detailed definitions and biological interpretations of all network metrics used). Edges represent statistical co-abundance associations; conclusions are restricted to network organization rather than directed biochemical regulation. Observed metrics were benchmarked against degree-preserving null networks to confirm nonrandom structure (empirical *P*-values, FDR-controlled). Networks were constructed using Spearman correlations (|*ρ*| ≥ 0.70; Benjamini–Hochberg FDR *q* < 0.05) via NetworkX v2.8.4. To verify that the findings were not dependent on the primary correlation threshold, the full network construction pipeline was repeated at |*ρ*| ∈ {0.60, 0.65, 0.70, 0.75, 0.80} (Benjamini–Hochberg FDR < 0.05) and at both VIP thresholds (≥1.0, ≥0.8), with the qualitative leaf–root asymmetry preserved across all 20 paired comparisons (Table S4; Fig. S8a to c). Community detection used the Louvain algorithm. Disconnected components were enumerated and classified by size; singleton nodes (degree 0) were cross-referenced against per-feature missingness in the preimputation abundance matrix (combined VIP ≥ 1.0 feature set; Table S8). Hub persistence and module preservation were validated through permutation testing (*n* = 10,000) with FDR correction. For fragmented networks, analyses focused on the largest connected component with size normalization. Hub overlap in Fig. 3a was calculated from the top-200 ranked hub-metabolite lists per network, capturing broad hub-list overlap; Table S5 separately reports a stricter top-20 core-hub Jaccard overlap between tissues across all VIP and correlation-threshold combinations.

Robustness assessment incorporated 3 approaches: (i) permutation testing (5,000 randomizations) with the Benjamini–Hochberg procedure; (ii) hub-connectivity pattern analysis using LOWESS smoothing (fraction = 0.3) (Cleveland 1979; Clauset et al. 2009); and (iii) tissue-specific network comparisons using nonparametric tests.

#### Temporal and cross-tissue network dynamics

Network dynamics were analyzed across 3 dimensions: temporal evolution, cross-tissue coordination, and stability. Stability of network-level metrics at *n* = 7 was quantified by cluster bootstrap resampling of replicate IDs (200 iterations) and leave-one-replicate-out jackknife, recomputing density, Louvain modularity *Q*, and LCC transitivity for each resample (Table S7; Fig. S9d to f). Cross-tissue coordination was quantified at each native time point as Spearman’s *ρ* between matched leaf and root median RI profiles, with leaf and root vectors joined by metabolite identity and restricted to a fixed shared metabolite set present across both genotypes and all sampled time points (*n* = 668 metabolites), ensuring that all temporal *ρ* values were computed from the same ordered feature set; 95% CIs were estimated by bootstrap resampling (*n* = 5,000). Network stability was analyzed separately: temporal patterns were assessed through nonparametric tests across sequential time points, and a sliding-window analysis (window = 3, step = 1) (Khan et al. 2010) tracked hub persistence and module preservation, with network coherence quantified via Kendall’s *W* and validated through permutation testing (*n* = 10,000) with FDR correction. For fragmented networks, analyses focused on the largest connected component with size normalization.

#### Bayesian network structure learning

Bayesian network structure was inferred using hill-climbing structure learning under a DAG constraint in bnlearn. Arc stability was estimated by nonparametric bootstrapping (5,000 resamples), retaining arcs with bootstrap strength ≥0.5. The primary analysis did not impose a maximum-parent constraint (maxp = Inf), yielding a maximum in-degree of 16 (leaf) and 13 (root). Departure from random structure was assessed using a permuted-data null in which metabolite columns were independently permuted across samples and the DAG was re-learned (5,000 permutations). Because maximum-parent caps are commonly used to control search complexity in high-dimensional structure learning, we additionally repeated structure learning under maxp ∈ {5, 3} and report arc counts, null expectations, and overlap with the unconstrained scaffold (Fig. S10; Table S10; Campos and Ji 2011). Parent constraints truncate high in-degree nodes by construction; Bayesian-network hubs (in-degree conditional dependencies) may therefore differ from Spearman hubs (undirected co-abundance degree), so we report both as complementary summaries of network organization.

Robustness assessment incorporated 3 sequential steps: (i) null model comparisons through permutation testing while preserving tissue-specific temporal structure; (ii) module preservation analysis using standardized preservation statistics and density scores; and (iii) cross-tissue comparisons using empirically derived *P*-values from bootstrap distributions.

### Cross-species validation in Arabidopsis

We accessed the public MetaboLights study MTBLS2289, a drought time-course in *Arabidopsis thaliana* (Lozano-Elena et al. 2022). We focused on contrasting genotypes (tolerant: 35S:BRL3-GFP; susceptible: bri1-301 bak1 brl1 brl3), retaining drought-treated shoot (rosette) and root samples (shoot *n* = 90, root *n* = 89). In the source study, aerial material comprises intact shoots harvested from 3-wk-old prebolting plants (rosette tissue), and individual leaf-rank separation was not performed (Lozano-Elena et al. 2022). Metabolites shared across tissues after quality filtering yielded 62 features retained for network construction. Initial preprocessing involved removing features with >30% missing values, median-imputing the remainder, and discarding zero-variance features. Abundances were then log1*p*-transformed and robustly standardized per metabolite (median/MAD with the 1.4826 consistency constant). Within each tissue, we computed pairwise Spearman correlations and constructed graphs where edges met 2 criteria applied in this order: Benjamini–Hochberg FDR *q* < 0.05 on the full pairwise matrix, then |*ρ*| ≥ 0.30. Thresholded graphs were treated as undirected and unweighted, with edge sign ignored for topology metrics. We calculated density, transitivity, mean shortest-path length (on the largest connected component), and modularity (*Q*) using the Louvain algorithm (*γ* = 1.0) with a fixed random seed. To assess nonrandomness, we generated 200 degree-preserving null surrogates for each empirical network via double-edge swaps (≈3×E swaps per surrogate). Departures from the nulls were summarized as *Z*-scores, *Z* = (*X*_obs_ −*μ*_null_)/*σ*_null_. For concise cross-tissue contrasts, we reported standardized differences, d^*^ = (shoot–root)/*σ*_pooled,null_, using the pooled standard deviation of the null distributions, with effect sizes interpreted using Cohen-style thresholds (0.2/0.5/0.8). The same pipeline was used to analyze tolerant and susceptible genotypes separately. Hubs were defined as the top-20 nodes by degree, and their overlap between genotypes was quantified using the Jaccard similarity. For roots, we characterized network rewiring trajectories by summarizing network density across Early (Days 1 to 2), Mid (3 to 4), and Late (5 to 6) windows. All analyses were performed in Python (NetworkX, SciPy). All stochastic steps were explicitly seeded (20250816). Complete outputs—including all reported summary values and sample counts—were serialized to a frozen lock file consumed by the plotting code, ensuring figure invariance and exact reproducibility.

### Software and statistical environment

All analyses were performed in Python 3.10 (using pandas, scikit-learn, NetworkX) and R 4.1.0 (using missForest, bnlearn, igraph). The complete preprocessing pipeline, custom analysis scripts, and data are publicly available on GitHub at: https://github.com/shoaibms/metabo.

## Supplementary Material

koag217_Supplementary_Data

## Data Availability

The processed metabolomics data, analysis scripts, and figures generated during this study are publicly available in a GitHub repository (https://github.com/shoaibms/metabo). The raw LC–MS data are publicly available in MetaboLights under accession MTBLS15125.
