# Supplementary material for: Conserved leaf–root metabolomic network asymmetry underpins divergent drought strategies
Source: Plant Cell. 2026 Jul 15;38(7):koag217. doi: 10.1093/plcell/koag217 (PMC13421925; doi:10.1093/plcell/koag217)
Supplement: koag217_Supplementary_Data [file koag217_supplementary_data.docx]

# Conserved leaf–root metabolomic network asymmetry underpins divergent drought strategies

**Authors**

Mirza Shoaib^1,2*^, Simone J Rochfort^2,3^, Priyanka Reddy^4^, Doris Ram^3^, Matthew J Hayden^2,3^ and Surya Kant^5^*

**Affiliations**

^1^Agriculture Victoria, Grains Innovation Park, 110 Natimuk Road, Horsham, Victoria 3400, Australia

^2^School of Applied Systems Biology, La Trobe University, 5 Ring Road, Bundoora, Victoria 3083, Australia

^3^Agriculture Victoria, AgriBio, Centre for AgriBioscience, 5 Ring Road, Bundoora, Victoria 3083, Australia

^4^ School of Chemistry, Bio21 Molecular Science and Biotechnology Institute, The University of Melbourne, 30 Flemington Road, Parkville,Victoria 3010, Australia

^5^Department of Ecological, Plant and Animal Science, School of Agriculture, Biomedicine & Environment, La Trobe University, 1 Kingsbury Drive, Bundoora, Victoria 3083, Australia

* Correspondence: [shoaib.mirza@agriculture.vic.gov.au](mailto:shoaib.mirza@agriculture.vic.gov.au) and [S.Kant@latrobe.edu.au](mailto:S.Kant@latrobe.edu.au)

*The author responsible for distribution of materials integral to the findings presented in this article, in accordance with the policy described in the Instructions for Authors, is Mirza Shoaib (shoaib.mirza@agriculture.vic.gov.au).*

# SUPPLEMENTARY INFORMATION

## General Supplementary Information

This document provides details about methods and findings that support the manuscript "Conserved leaf–root metabolomic network asymmetry underpins divergent drought strategies." The methods section outlines protocols for metabolite identification, including database matching, structural characterisation, and pathway mapping. The results section presents analyses of responses to stress, featuring profiling of groups, analyses across genotypes, and visualisations that complement findings. These materials include figures, analyses, and tables that show the approach and provide context for metabolic adaptations in wheat under stress conditions.

The following Supporting Information is available for this article:

Table S1: Network analysis terminology

| **Network Metric / Concept** | **Definition (General Network Science)** | **Conceptual Calculation / Underlying Logic** | **General Biological Meaning/Interpretation** | **Relevant Reference(s) for Definition & Biological Interpretation** |
| --- | --- | --- | --- | --- |
| **Network Density** | The proportion of existing connections (edges) in a network is relative to the total number of possible connections. | Logic: Measures how “full” or “sparse” a network is. Calculated as 2E / (N(N-1)) for undirected networks or E / (N(N-1)) for directed, where E is edges, N is nodes. Ranges from 0 (no connections) to 1 (fully connected). Most biological networks have low density (< 0.1) due to metabolic constraints. | Biologically, high density can suggest a system with many interactions, potentially leading to faster signal propagation, robustness to loss of individual connections, or higher functional redundancy. Low density might imply specialisation or efficiency with fewer links. | (Erdos and Rényi, 1960); Barabási and Oltvai (2004); (Grafahrend-Belau et al., 2009) |
| **Transitivity (Global Clustering Coefficient)** | A measure of the overall tendency for nodes in a network to cluster together. Specifically, it quantifies the likelihood that two neighbors of a given node are themselves connected. | Logic: Assesses the “cliquishness” of a network. Calculated as 3 * (number of triangles) / (number of connected triplets of nodes). A triangle is three nodes all connected to each other. A connected triplet is a node connected to two others. Ranges from 0 to 1. Values > 0.3 typically indicate significant clustering in biological networks. | High transitivity often indicates the presence of tightly coupled functional modules or pathways where components work closely together. It can contribute to network robustness and efficient local information processing. | (Watts and Strogatz, 1998); Barabási and Oltvai (2004) |
| **Modularity (Q)** | A measure of the strength of division of a network into modules (also called groups, clusters, or communities). Networks with high modularity have dense connections between the nodes within modules but sparse connections between nodes in different modules. | Logic: Quantifies how well a network can be partitioned into non-overlapping communities. Algorithms (e.g., Louvain) optimise Q by comparing the fraction of edges within communities to the expected fraction if edges were random. Higher Q indicates stronger community structure. Ranges from -0.5 to 1. Values > 0.3 generally indicate strong community structure. Biological networks typically show Q = 0.3-0.7. | Biologically, modularity often reflects functional specialisation. Different modules might correspond to distinct biological pathways, complexes, or processes that can operate somewhat independently, allowing for adaptability and robustness. | (Girvan and Newman, 2002; Barabási and Oltvai, 2004); Newman (2006); (Fortunato, 2010) |
| **Mean Path Length** | The average number of edges along the shortest paths connecting all possible pairs of nodes in a network. | Logic: Measures the typical separation between any two nodes, reflecting the efficiency of information or mass transfer across the network. Calculated by finding all the shortest paths, summing their lengths, and dividing by the total number of pairs. | Short mean path lengths are characteristic of “small-world” networks and imply efficient communication or rapid propagation of signals throughout the system. This can be crucial for coordinated responses. | Watts and Strogatz (1998); (Latora and Marchiori, 2001; Barabási and Oltvai, 2004) |
| **Degree Centrality (Node Degree)** | The number of direct connections (edges) a node has to other nodes in the network. | Logic: A simple, local measure of a node’s direct influence or activity within the network. Count the number of edges incident to a node. | Nodes with high degree (hubs) are often critical for network function and stability. They can act as control points, integrate signals, or be essential components of many pathways. Loss of hubs can be highly disruptive. | (Jeong et al., 2001); Barabási and Oltvai (2004); (Clauset et al., 2009) |
| **Network Components (Connected Components)** | Subgraphs in which any two nodes are connected to each other by paths, and which are connected to no additional nodes in the supergraph. A network can consist of one or multiple components. | Logic: Identifies disconnected parts of a network. Algorithms like Breadth-First Search or Depth-First Search can identify all nodes reachable from a starting node, thus defining a component. | The number and size of components indicate the overall cohesiveness of a network. A single large component suggests an integrated system. Multiple components might suggest independent functional units or fragmentation due to stress or missing data. | (Erdos and Rényi, 1960; Newman, 2010) |
| **Assortativity (Degree Assortativity)** | A measure of the preference for nodes in a network to attach to other nodes that are similar (assortative) or dissimilar (disassortative) in terms of their degree. | Logic: Assesses correlation between the degrees of connected nodes. Calculated as the Pearson correlation coefficient of the degrees at either end of every edge. Positive values indicate assortativity; negative values indicate disassortativity. Most biological networks are disassortative (negative values). | Biological networks are often disassortative (hubs connect to low-degree nodes), which can make them robust to random failures but vulnerable to targeted attacks on hubs. It can also reflect hierarchical organisation. | Newman (2002) |
| **Network Stability/Robustness** | The ability of a network to maintain its essential structural properties and functions despite perturbations (e.g., node/edge removal, changes over time). | Logic: Assessed through various means, e.g., measuring how key metrics (density, path length, size of largest component) change after simulated perturbations, or how module structures persist across different states (module preservation). | High stability/robustness is crucial for biological systems to function reliably in fluctuating environments or under stress. It reflects the system’s resilience and capacity to maintain homeostasis or adapt effectively. | Albert et al. (2000); (Fait et al., 2020) |
| **Hub Distribution/Connectivity Pattern** | The way high-degree nodes (hubs) are distributed across the network - either concentrated (few very high-degree nodes) or distributed (more evenly spread connectivity). | Logic: Analyse the degree distribution curve, often through rank-degree plots or power-law fitting. Concentrated = steep decay; Distributed = gradual decay. Can be quantified through measures like the Gini coefficient of degree distribution. | Concentrated hubs provide centralised control points for rapid coordination (like in leaves for photosynthetic responses). Distributed hubs allow for more flexible, decentralised responses (like roots adapting to soil heterogeneity). | Barabási and Albert (1999); (Jeong et al., 2001; Clauset et al., 2009) |
| **Temporal Coherence** | A measure of how consistently connected nodes in a network respond together over time. | Logic: Often calculated as correlation of responses between connected nodes across time points, or using metrics like Kendall’s W for concordance. Can also be measured as the stability of correlation patterns over time. | High temporal coherence suggests coordinated responses within functional modules. Low coherence may indicate independent responses or system fragmentation under stress. Essential for understanding dynamic adaptation strategies. | Fait et al. (2020) |
| **Cross-tissue Correlation** | The degree of similarity in metabolic responses between different tissues (e.g., leaf vs. root) over time. | Logic: Correlation analysis (e.g., Spearman correlation) of metabolite profiles between tissues, often tracked over multiple time points. Can reveal coordination patterns and declining cross-tissue coordination. | High cross-tissue correlation suggests coordinated whole-plant responses. Declining cross-tissue coordination (decreasing correlation) may indicate tissue-specific adaptation strategies. Critical for understanding organ-level coordination. | Gargallo-Garriga et al. (2014) |
| **Module Preservation** | The extent to which network modules (communities) maintain their structure across different conditions or time points. | Logic: Statistical measures comparing module membership between conditions, often using preservation statistics like those in WGCNA (Weighted Gene Co-expression Network Analysis). Involves permutation testing and standardised preservation scores. | High module preservation indicates stable functional organisation. Dynamic module reorganisation can reflect adaptive responses to environmental changes. Important for understanding functional stability under stress. | (Zhang and Horvath, 2005; Fortunato, 2010); Langfelder et al. (2011) |
| **Network Motifs** | Recurring patterns of interconnections that occur in networks at numbers that are significantly higher than those in randomised networks. | Logic: Identified by comparing subgraph frequencies in real networks vs. randomised null models. Common motifs include feedforward loops, feedback loops, and bi-fans. Statistical significance assessed through Z-scores. | Network motifs represent fundamental building blocks of biological networks and often correspond to basic regulatory or metabolic functions. They provide insights into evolutionary constraints and functional organisation. | (Barabási and Oltvai, 2004); Alon (2007) |

Table S2. Nutrient solution used in the controlled-environment hydroponic experiments.

| **No** | **Nutrient** |  | | **Concentration** |
| --- | --- | --- | --- | --- |
| 1 | MgSO_4_ |  |  | 4 mM |
| 2 | CaCl_2_.2H_2_O |  |  | 4 mM |
| 3* | KH_2_PO_4_ pool |  |  | 3 mM |
|  | K_2_HPO_4_ pool |  |  |  |
| 4 | Fe^+^ (FeEDTA) |  |  | 400 µM |
| 5 | MnCl_2_.4H_2_O |  |  | 10 µM |
|  | ZnSO_4_.7H_2_O |  |  | 10 µM |
|  | CuSO_4_ |  |  | 2 µM |
|  | H_3_BO_3_ |  |  | 50 µM |
|  | Na_2_MoO_4_ |  |  | 0.2 µM |
| 6 | KNO_3_ |  |  | 1M |

*Pooled to make pH 6

Materials and Methods Details
Data Processing Parameters

Table S3. Genedata setting for quantitation

| Processing Step | Parameter | Positive Mode | Negative Mode |
| --- | --- | --- | --- |
| Data Import | RT Range | 0-16 minutes | 0-16 minutes |
| Chromatogram Chemical Noise Subtraction | Smoothing RT Window | 3 scans | 3 scans |
|  | Subtraction RT Window | 51 scans | 51 scans |
|  | Quantile | 50% | 50% |
|  | Intensity Threshold | 8000, 10000 | 3000 |
| RT Alignment | Max RT Shift | 0.4 minutes | 0.2 minutes |
|  | Gap Penalty | 1 | 1 |
| Peak Detection | Summation Window | 5 scans | 5 scans |
|  | Minimum Peak Size | 3 scans | 4 scans |
|  | Maximum Merge Distance | 5 points | 5 points |
|  | Merge Strategy | Centres | Boundaries |
|  | Smoothing | m/z window: 3 points | m/z window: 3 points |
|  | Refinement Threshold | 5% | 5% |
|  | Consistency Threshold | 0.6 | 0.6 |
| Isotope Clustering | RT Tolerance | 0.05 minutes | 0.05 minutes |
|  | m/z Tolerance | 5 ppm | 5 ppm |
|  | Ionisation | Protonation | Deprotonation |
|  | Charge Range | 1–2 | 1-5 |
|  | Distance Measure | Log-Ratio | Log-Ratio |
|  | Max Distance | 0.5 | 0.5 |

Supplementary Table S4. Network architecture robustness across analytical thresholds.

| vip_threshold | rho_threshold | tissue | genotype | graph_edges | density | modularity_louvain | transitivity | lcc_frac |
| --- | --- | --- | --- | --- | --- | --- | --- | --- |
| 0.8000 | 0.6000 | Leaf | G1 | 297063 | 0.4690 | 0.0747 | 0.8636 | 0.9591 |
| 0.8000 | 0.6000 | Leaf | G2 | 328721 | 0.5190 | 0.1011 | 0.8578 | 0.9707 |
| 0.8000 | 0.6000 | Root | G1 | 206383 | 0.2869 | 0.2634 | 0.7403 | 0.9550 |
| 0.8000 | 0.6000 | Root | G2 | 216500 | 0.3009 | 0.2654 | 0.7555 | 0.9583 |
| 0.8000 | 0.6500 | Leaf | G1 | 257421 | 0.4064 | 0.0858 | 0.8401 | 0.9334 |
| 0.8000 | 0.6500 | Leaf | G2 | 285766 | 0.4512 | 0.1186 | 0.8319 | 0.9476 |
| 0.8000 | 0.6500 | Root | G1 | 163702 | 0.2276 | 0.2801 | 0.7155 | 0.9292 |
| 0.8000 | 0.6500 | Root | G2 | 172171 | 0.2393 | 0.2971 | 0.7339 | 0.9358 |
| 0.8000 | 0.7000 | Leaf | G1 | 212388 | 0.3353 | 0.1014 | 0.8058 | 0.8970 |
| 0.8000 | 0.7000 | Leaf | G2 | 236402 | 0.3732 | 0.1449 | 0.7972 | 0.9201 |
| 0.8000 | 0.7000 | Root | G1 | 121779 | 0.1693 | 0.3142 | 0.6860 | 0.8758 |
| 0.8000 | 0.7000 | Root | G2 | 129928 | 0.1806 | 0.3369 | 0.7057 | 0.9075 |
| 0.8000 | 0.7500 | Leaf | G1 | 161163 | 0.2545 | 0.1209 | 0.7620 | 0.8472 |
| 0.8000 | 0.7500 | Leaf | G2 | 179981 | 0.2842 | 0.1809 | 0.7513 | 0.8659 |
| 0.8000 | 0.7500 | Root | G1 | 81784 | 0.1137 | 0.3607 | 0.6519 | 0.8275 |
| 0.8000 | 0.7500 | Root | G2 | 89422 | 0.1243 | 0.3883 | 0.6710 | 0.8725 |
| 0.8000 | 0.8000 | Leaf | G1 | 106492 | 0.1681 | 0.1549 | 0.7043 | 0.7993 |
| 0.8000 | 0.8000 | Leaf | G2 | 118214 | 0.1866 | 0.2293 | 0.6878 | 0.8135 |
| 0.8000 | 0.8000 | Root | G1 | 47457 | 0.0660 | 0.4301 | 0.6167 | 0.7692 |
| 0.8000 | 0.8000 | Root | G2 | 54701 | 0.0760 | 0.4579 | 0.6459 | 0.8117 |
| 1.0000 | 0.6000 | Leaf | G1 | 36449 | 0.3624 | 0.0722 | 0.8527 | 0.9131 |
| 1.0000 | 0.6000 | Leaf | G2 | 46658 | 0.4639 | 0.0939 | 0.8622 | 0.9354 |
| 1.0000 | 0.6000 | Root | G1 | 46514 | 0.2606 | 0.2647 | 0.7489 | 0.9498 |
| 1.0000 | 0.6000 | Root | G2 | 49253 | 0.2759 | 0.2917 | 0.7374 | 0.9615 |
| 1.0000 | 0.6500 | Leaf | G1 | 31074 | 0.3090 | 0.0777 | 0.8386 | 0.8731 |
| 1.0000 | 0.6500 | Leaf | G2 | 40477 | 0.4025 | 0.1099 | 0.8425 | 0.9042 |
| 1.0000 | 0.6500 | Root | G1 | 37162 | 0.2082 | 0.2889 | 0.7345 | 0.9197 |
| 1.0000 | 0.6500 | Root | G2 | 39107 | 0.2191 | 0.3271 | 0.7197 | 0.9415 |
| 1.0000 | 0.7000 | Leaf | G1 | 25478 | 0.2533 | 0.0903 | 0.8114 | 0.7996 |
| 1.0000 | 0.7000 | Leaf | G2 | 33817 | 0.3362 | 0.1308 | 0.8163 | 0.8686 |
| 1.0000 | 0.7000 | Root | G1 | 28265 | 0.1583 | 0.3197 | 0.7155 | 0.8729 |
| 1.0000 | 0.7000 | Root | G2 | 29798 | 0.1669 | 0.3710 | 0.7025 | 0.9080 |
| 1.0000 | 0.7500 | Leaf | G1 | 19259 | 0.1915 | 0.1074 | 0.7755 | 0.7483 |
| 1.0000 | 0.7500 | Leaf | G2 | 26274 | 0.2612 | 0.1617 | 0.7763 | 0.7973 |
| 1.0000 | 0.7500 | Root | G1 | 19890 | 0.1114 | 0.3610 | 0.6857 | 0.8278 |
| 1.0000 | 0.7500 | Root | G2 | 20922 | 0.1172 | 0.4274 | 0.6863 | 0.8712 |
| 1.0000 | 0.8000 | Leaf | G1 | 12939 | 0.1286 | 0.1489 | 0.7226 | 0.6459 |
| 1.0000 | 0.8000 | Leaf | G2 | 17739 | 0.1764 | 0.2048 | 0.7237 | 0.7550 |
| 1.0000 | 0.8000 | Root | G1 | 12432 | 0.0696 | 0.4223 | 0.6473 | 0.7625 |
| 1.0000 | 0.8000 | Root | G2 | 13356 | 0.0748 | 0.4889 | 0.6755 | 0.7860 |

Supplementary Table S5. Leaf-root hub identity overlap (Jaccard index) across thresholds**.**

| vip_threshold | rho_threshold | genotype | hub_jaccard_leaf_vs_root |
| --- | --- | --- | --- |
| 0.8000 | 0.6000 | G1 | 0.0000 |
| 0.8000 | 0.6000 | G2 | 0.0000 |
| 0.8000 | 0.6500 | G1 | 0.0256 |
| 0.8000 | 0.6500 | G2 | 0.0000 |
| 0.8000 | 0.7000 | G1 | 0.0256 |
| 0.8000 | 0.7000 | G2 | 0.0000 |
| 0.8000 | 0.7500 | G1 | 0.0256 |
| 0.8000 | 0.7500 | G2 | 0.0000 |
| 0.8000 | 0.8000 | G1 | 0.0526 |
| 0.8000 | 0.8000 | G2 | 0.0000 |
| 1.0000 | 0.6000 | G1 | 0.0000 |
| 1.0000 | 0.6000 | G2 | 0.0000 |
| 1.0000 | 0.6500 | G1 | 0.0256 |
| 1.0000 | 0.6500 | G2 | 0.0256 |
| 1.0000 | 0.7000 | G1 | 0.0256 |
| 1.0000 | 0.7000 | G2 | 0.0000 |
| 1.0000 | 0.7500 | G1 | 0.0256 |
| 1.0000 | 0.7500 | G2 | 0.0000 |
| 1.0000 | 0.8000 | G1 | 0.0526 |
| 1.0000 | 0.8000 | G2 | 0.0000 |

Supplementary Table S6. Non-parametric statistical power by comparison type.

| comparison_type | n_comparisons | power_mean | power_median | power_sd | pct_power_ge_0.80 |
| --- | --- | --- | --- | --- | --- |
| tissue | 8508 | 0.5670 | 0.6393 | 0.3323 | 38.31 |
| genotype | 18834 | 0.1192 | 0.0794 | 0.1004 | 0.0000 |
| temporal | 12556 | 0.0471 | 0.0458 | 0.0063 | 0.0000 |

Supplementary Table S7. Network metric stability under cluster-bootstrap resampling.

| tissue | genotype | metric | baseline | bootstrap_median | bootstrap_ci_lo | bootstrap_ci_hi | jackknife_cv |
| --- | --- | --- | --- | --- | --- | --- | --- |
| Leaf | G1 | density_all | 0.2369 | 0.2341 | 0.1578 | 0.3334 | 0.0736 |
| Leaf | G1 | modularity_all | 0.2131 | 0.2355 | 0.1437 | 0.3378 | 0.0986 |
| Leaf | G1 | transitivity_lcc | 0.7373 | 0.7223 | 0.6423 | 0.7857 | 0.0245 |
| Leaf | G2 | density_all | 0.2255 | 0.2321 | 0.1524 | 0.2953 | 0.0745 |
| Leaf | G2 | modularity_all | 0.2536 | 0.2546 | 0.1964 | 0.3586 | 0.0703 |
| Leaf | G2 | transitivity_lcc | 0.6974 | 0.6974 | 0.6128 | 0.7562 | 0.0245 |
| Root | G1 | density_all | 0.1477 | 0.1592 | 0.1319 | 0.1990 | 0.0599 |
| Root | G1 | modularity_all | 0.2976 | 0.2956 | 0.2266 | 0.3821 | 0.0714 |
| Root | G1 | transitivity_lcc | 0.7174 | 0.7098 | 0.6487 | 0.7752 | 0.0261 |
| Root | G2 | density_all | 0.1494 | 0.1562 | 0.1160 | 0.2506 | 0.0901 |
| Root | G2 | modularity_all | 0.3666 | 0.3517 | 0.2446 | 0.4306 | 0.0807 |
| Root | G2 | transitivity_lcc | 0.6941 | 0.6899 | 0.6471 | 0.7354 | 0.0078 |

Supplementary Table S8. Singleton node audit: pre-imputation missingness cross-reference.

| tissue | genotype | n_singletons | lcc_frac | isolates_median_missing_pct | connected_median_missing_pct | n_all_na_dropped |
| --- | --- | --- | --- | --- | --- | --- |
| Leaf | G1 | 51 | 0.8314 | 0.0000 | 0.0000 | 0 |
| Leaf | G2 | 39 | 0.8669 | 0.0000 | 0.0000 | 0 |
| Root | G1 | 78 | 0.8531 | 0.0000 | 0.0000 | 0 |
| Root | G2 | 57 | 0.8956 | 0.0000 | 0.0000 | 0 |

Supplementary Table S9. Concordance between ANOVA and Mann-Whitney U test.

| scope | n_comparisons_total | pct_passing_normality | pct_concordant | n_discordant_anova_only_sig | n_discordant_mw_only_sig |
| --- | --- | --- | --- | --- | --- |
| Leaf | 8508 | 25.31 | 94.19 | 121 | 4 |
| Root | 10326 | 45.30 | 91.73 | 381 | 6 |
| Overall | 18834 | 36.27 | 92.50 | 502 | 10 |

Supplementary Table S10. Bayesian network topology under maximum-parent constraints.

| dataset | maxp | n_bootstraps | observed_edges | kept_edges_bs_ge_0.5 | max_in_degree | null_edges_mean | null_edges_sd | p_perm | overlap_with_unconstr |
| --- | --- | --- | --- | --- | --- | --- | --- | --- | --- |
| Leaf | inf | 5000 | 493 | 109 | 16 | 105.60 | 11.10 | 0.0002 | NA |
| Leaf | 5.0000 | 1000 | 344 | 79 | 5 | 103.80 | 11.00 | 0.0010 | 0.9160 |
| Leaf | 3.0000 | 1000 | 229 | 71 | 3 | 97.10 | 9.4000 | 0.0010 | 0.8860 |
| Root | inf | 5000 | 406 | 121 | 13 | 105.90 | 11.20 | 0.0002 | NA |
| Root | 5.0000 | 1000 | 313 | 96 | 5 | 104.80 | 10.60 | 0.0010 | 0.9270 |
| Root | 3.0000 | 1000 | 220 | 90 | 3 | 96.80 | 9.4000 | 0.0010 | 0.9050 |

### Detailed Data Preprocessing Workflow

#### Initial Data Cleaning

We first excluded columns containing fewer than three replicates for each molecular feature. Despite this reduction, missing values persisted, a common issue in LC-MS studies (Karpievitch et al., 2012) (Kokla et al., 2019). We assessed the nature of the missing data using Little's MCAR test and logistic regression to determine whether they were Missing Completely at Random (MCAR), Missing at Random (MAR), or Missing Not at Random (MNAR).

#### Missing Value Analysis and Imputation

We employed a diverse set of imputation techniques, including machine learning (Wei et al., 2018), neighbour-based methods, and matrix factorisation. In R, we used Random Forest (RF) (maxiter = 5, ntree = 50) and Predictive Mean Matching (PMM), while in Python, we applied Bayesian PCA, k-Nearest Neighbours (KNN), SVD, Gaussian Process Regression (GPR), and Expectation-Maximisation (EM). The performance of these methods was evaluated using Earth Mover's Distance (EMD) and Hellinger Distance, alongside visual comparisons via Q-Q plots, ECDF plots, and KDE plots. Additionally, we calculated richness, Shannon entropy, Simpson's diversity index, and sparsity for datasets imputed with median and RF methods.

#### Outlier Detection, Removal

Post-imputation, we numerically encoded categorical variables and standardised molecular features. Seven outlier detection techniques were applied: Mahalanobis Distance, Isolation Forest, Elliptic Envelope, Robust PCA, Local Outlier Factor, Z-Score, and IQR. We assessed the effectiveness of these methods by quantifying outliers and adjusting contamination thresholds, with visualisation via PCA and t-SNE plots. Outliers identified were removed, and resulting NaN values were imputed using the validated Random Forest method (missForest package). The 30 most affected variables were plotted with standard deviation error bars to highlight changes after outlier removal and imputation.

#### Data Transformation and Variable Selection

Following outlier removal, various data transformations were applied: Log, Square Root, Box-Cox, Yeo-Johnson, Generalised Hyperbolic Sine (asinh) (using its standard mathematical definition, asinh(x) = ln(x + sqrt(x^2 + 1)), Generalised Logarithmic (glog), and Anscombe. We assessed their impact on data variability and distribution using Coefficient of Variation (CV), MA-transform (log ratio M), Relative Standard Deviation (RSD), and Relative Median Absolute Deviation (rMAD). Normality was evaluated using Shapiro-Wilk and Anderson-Darling tests, with density plots visualising the distribution of test statistics and p-values for each variable and transformation method.


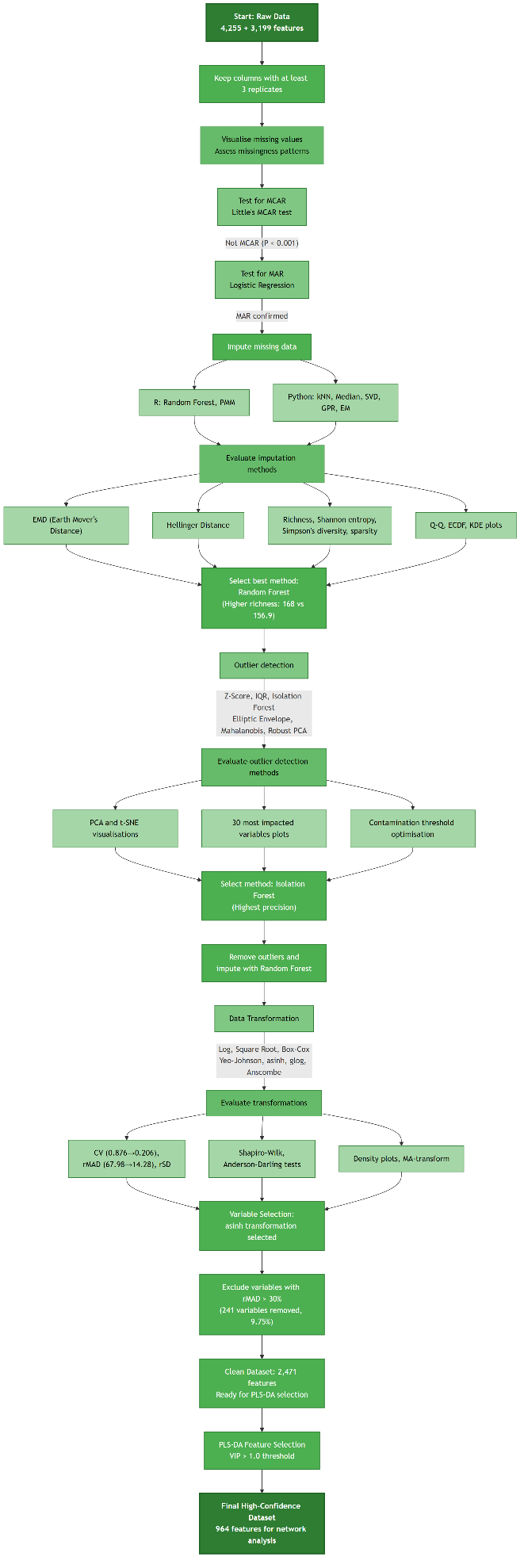


Fig. S1. Data preprocessing steps. Supports Fig. 1 (Methods): documents the preprocessing pipeline that produced the 2,471-feature dataset underlying all main-text networks.

### Metabolite Annotation Methods

#### Database Matching (HMDB)

Untargeted metabolomics data was matched against the Human Metabolome Database (HMDB) using a custom Python script. The script first extracted the HMDB data from a compressed XML file, parsing key metabolite properties including chemical identifiers (InChI, InChIKey, SMILES), structural information (formula, super class, sub class), biological context (pathways, tissue locations), and database cross-references (KEGG, PubChem, Metlin IDs). For metabolite matching, the script prioritised matching by exact mass when available, with m/z as a fallback, applied a 10 ppm mass tolerance filter, selected the match with the smallest ppm difference for each cluster, and calculated quality metrics including m/z rank (normalised mass difference) and RT rank (normalised retention time difference). The results were exported as separate CSV files for negative and positive ionisation modes, containing comprehensive metabolite annotations and match quality assessments. The script handled missing values gracefully and included extensive error checking to ensure data integrity.

$$m/z Rank=\frac{[Experimental m/z-HMDB Exact mass]}{Experimental m/z}$$

$$RT Rank=\frac{[Experimental RT-HMDB RT]}{Maximum RT in Dataset}$$

#### GNPS-Based Annotation Enhancement

The metabolite matches were further annotated using the GNPS database API. The script queried GNPS using chemical identifiers (InChIKey, SMILES, or PubChem CID) to retrieve additional chemical classifications, pathways, and biospecimen data. The data were then enhanced with tissue-type annotations by mapping metabolite clusters to their corresponding root or leaf tissue origins. Finally, the script integrated statistical metrics from VIP and Mann-Whitney analyses, creating comprehensive annotation files for both ionisation modes. All results were exported as CSV files with complete metabolite characterisation and tissue distribution information.

#### Structural Analysis using RDKit

The putatively identified metabolites underwent comprehensive structural characterisation using RDKit through two complementary approaches: structural classification and functional group analysis. In the classification phase, each metabolite's SMILES structure was evaluated against curated SMARTS patterns representing both broad biomolecule classes (like lipids and carbohydrates) and specific plant compounds (such as phytohormones). A confidence scoring system assessed matches based on molecular pattern complexity and atomic substructure matching, with weighted priorities resolving overlapping classifications. The functional group analysis then systematically identified 30 key structural features (including hydroxyls, carboxyls, and aromatic rings) using parallel processing for computational efficiency. The analyses enriched the metabolite annotations with structural classifications, quantitative confidence scores (0-1), and detailed functional group profiles.

#### Pathway Mapping

We performed metabolic pathway analysis of stress responses using non-parametric statistics. Metabolite changes between control and stress conditions were assessed using Mann-Whitney U tests, with false discovery rate (FDR) correction (Benjamini-Hochberg) applied within each tissue-genotype-time points-batch combination. Significant changes were defined by FDR < 0.05 and minimum three replicates per condition. Median fold changes and 95% confidence intervals were calculated using bootstrap resampling (1000 iterations). These significant metabolite changes were then mapped to KEGG pathways to assess pathway-level responses, considering both the number and magnitude of metabolite changes within each pathway. This approach provided a comprehensive view of metabolic pathway modulation under stress conditions.

## Supplementary Results

### Data Preprocessing Results

#### Initial Data Cleaning Outcomes

Initial data filtering reduced the number of molecular features from 4,255 to 1,789 in one dataset and from 3,199 to 1,350 in another, by excluding entries with fewer than three replicates. Little’s MCAR test did not reject the hypothesis that missing values were completely at random (P = 1.0). Logistic-regression analyses were also used to examine associations between missingness and observed experimental variables, including genotype, treatment, time point, and replicate (Fig. S2A). Heatmaps (Fig. S2B-C) illustrate missing value patterns in leaf and root datasets for negative columns, with missing data in green and non-missing in grey.

#### Missing Value Patterns and Imputation Performance

The stacked bar plot of Earth Mover's Distance (Fig. S2D) indicated that median imputation performed well overall, closely followed by Random Forest (RF). Hellinger Distance (Fig. S2E) supported these findings. However, RF imputation outperformed median imputation in key metrics, with higher richness (168 vs. 156.9), Shannon entropy (5.124 vs. 4.999), and Simpson's diversity index (0.994 vs. 0.992). RF also demonstrated lower sparsity (0.00595 vs. 0.0204), preserving more variability.

Graphical comparisons, including Q-Q plots (Fig. S2F, left panel), ECDF, and KDE plots (Fig. S2F, middle and right panels), showed that RF imputation more closely aligned with the original data distribution. Based on these visual and statistical evaluations, RF was selected as the optimal imputation method.

#### Outlier Analysis Results

Isolation Forest emerged as the most effective outlier detection method, as visualised through PCA and t-SNE plots (Fig. S2g, h) and supported by the analysis of the 30 most impacted variables. This method identified outliers with the highest precision, leading to its selection for further analysis.

#### Transformation Effects

The asinh transformation most effectively reduced data variability, decreasing CV from 0.876 to 0.206 (Fig. S2I), rMAD from 67.98 to 14.28 (Fig. S2J), and RSD from 87.36% to 20.58% (Fig. S2K). MA-transformation plots confirmed tighter data clustering (Fig. S2L, M). Normality improved moderately across transformations, with asinh offering the best balance (Fig. S2N-Q). However, most variables remained non-parametric, guiding subsequent analysis choices.

In negative mode, the leaf dataset contained 807 molecular features, and the root dataset contained 982, with 391 features shared between tissues, resulting in a combined total of 1,398 molecular features. In positive mode, the leaf dataset contained 611 molecular features, and the root dataset contained 739, with 277 features shared between tissues, resulting in a combined total of 1,073 molecular features. Overall, the combined dataset comprised 2,471 molecular features.


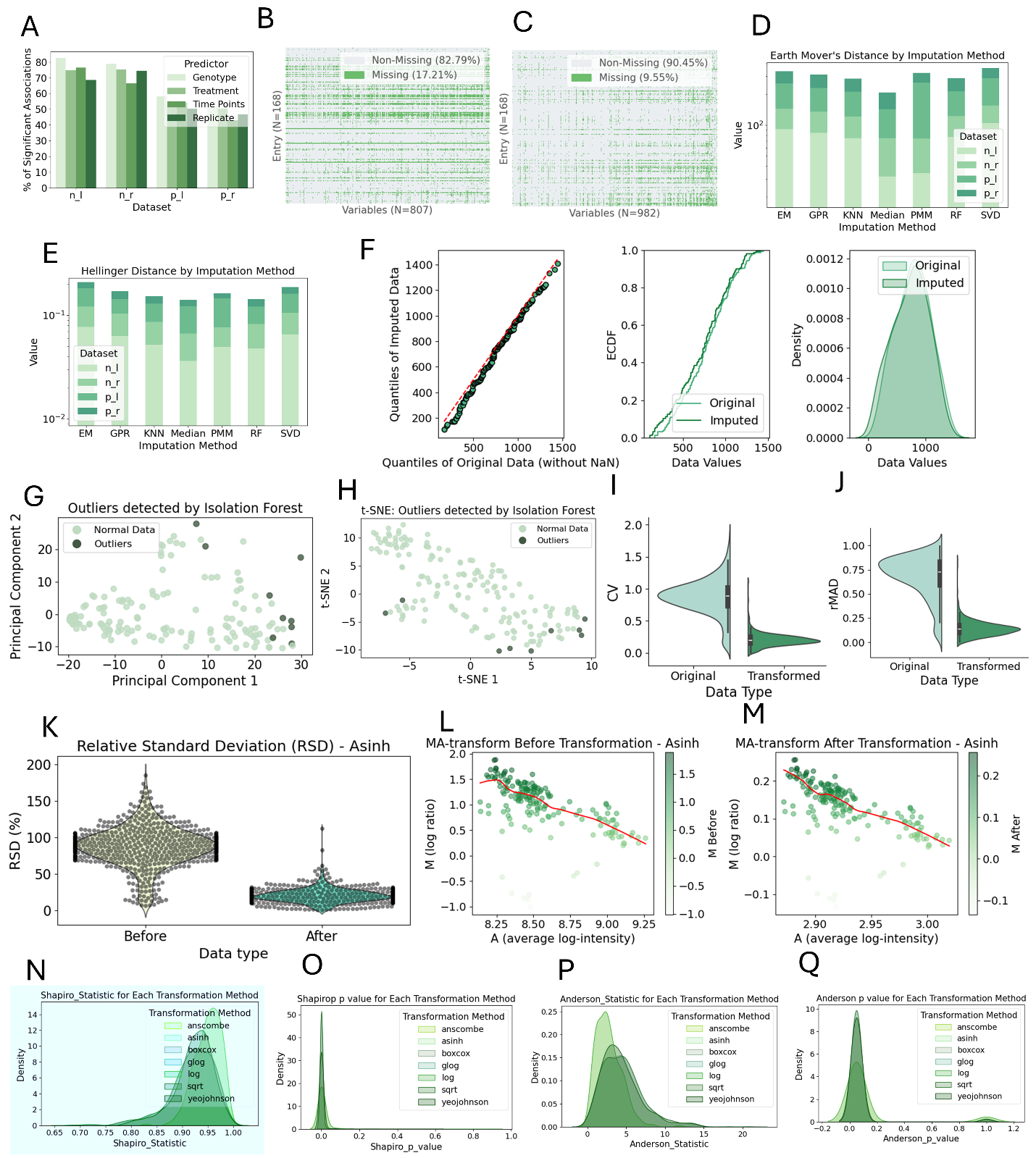


Fig. S2. Data Preprocessing and Evaluation. Supports Fig. 1 (Methods): validates the missing-data handling and imputation underlying all main-text analyses.

A, Logistic-regression analysis of missing-data patterns. Bar chart summarising associations between missingness and genotype, treatment, time point, and replicate across datasets. B, Missing Value Patterns. Heatmaps depicting missing (green) and non-missing (grey) data distribution in negative mode leaf (n_l) B and root (n_r) C, datasets. D-E, Imputation Method Comparison. Stacked bar plots of Earth Mover's Distance D and Hellinger Distance E for various imputation methods across all datasets, highlighting the superior performance of RF and median imputation. F, Graphical Evaluation of Imputation Methods. Q-Q, ECDF, and KDE plots comparing original and RF-imputed data distributions across all datasets, showing that RF-imputed data closely aligns with the original distribution. G-H, PCA and t-SNE plots of the n_l dataset. These plots show outliers (dark points) at the periphery of clustered normal data (light green), demonstrating Isolation Forest's effectiveness in identifying anomalies across different dimensions. I-J, Data Transformation Effects. Density plots showing the impact of asinh transformation on Coefficient of Variation (CV) I and Relative Median Absolute Deviation (rMAD) J. K Relative Standard Deviation (RSD) change. Violin plot demonstrating RSD reduction in the negative mode leaf dataset (n_l). L-M, MA-Transform Plots. Scatter plots illustrating the distribution of the n_l dataset before L and after M asinh transformation. N-Q, Normality Assessment. Density plots of Shapiro-Wilk N,O and Anderson-Darling P,Q test statistics and p-values for various transformation methods on the n_l dataset.

### Metabolite Analysis Results

#### Tissue-specific Temporal Adjustments

Temporal analysis revealed distinct response patterns between leaf and root tissues under osmotic stress. Leaf tissue exhibited rapid metabolic adjustments at the initial timepoint, while root tissue showed a delayed response peaking at the second timepoint. This tissue-specific temporal divergence was evident across multiple chemical classes and functional groups, with leaves showing coordinated early responses and roots displaying more gradual adaptation patterns. Notably, thiol, a key osmotic stress marker, was exclusively detected in root tissue. These tissue-specific metabolic dynamics are visualised in Fig. S3–S6, which detail the temporal progression of chemical groups, functional responses, and pathway-level changes across tissues and genotypes. The figures illustrate how these coordinated metabolic adjustments contribute to the distinct network architectures observed in the main manuscript, highlighting the sophisticated regulation of both broad chemical classes and specific metabolic pathways during stress adaptation.

### Supplementary Figures


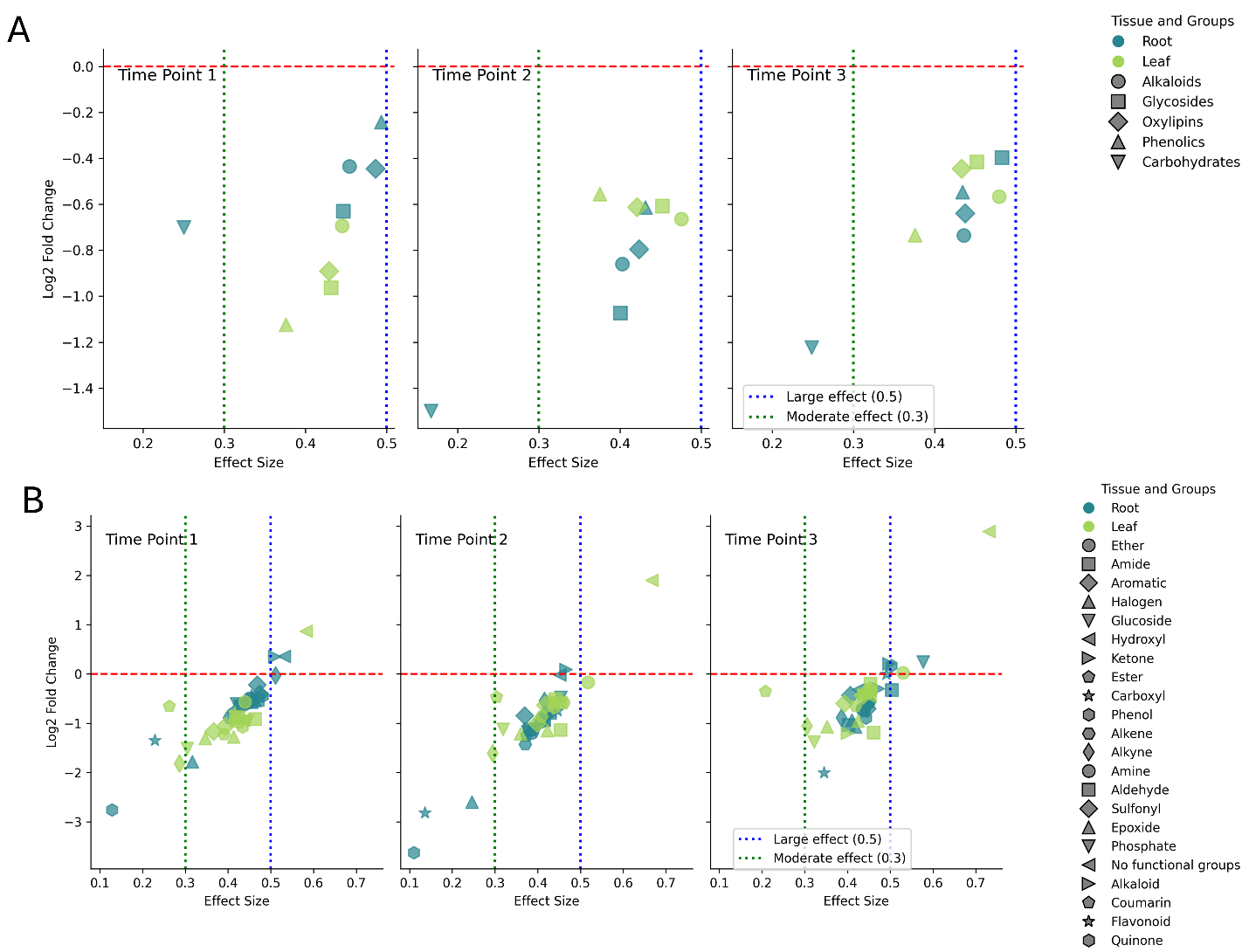

Fig. S3. Temporal dynamics of chemical and functional group responses to osmotic stress across tissues. Supports Fig. 2 and Fig. 3: provides the chemical- and functional-group basis for the tissue-specific temporal responses summarised in the main text.

The temporal progression reveals tissue-specific patterns of chemical (A) and functional (B) groups. In chemical groups (A), leaf tissue demonstrates a rapid response with maximum decreases in metabolites at Time Point 1, while root tissue shows delayed responses peaking at Time Point 2. Most functional groups (B) mirror this tissue-specific temporal pattern, with leaves showing early responses (Time Point 1) and roots exhibiting delayed but sustained modulation across Time Points 2 and 3. Effect sizes are demarcated by vertical dotted lines indicating moderate (0.3, green) and large (0.5, blue) effects. Log2 fold changes quantify the magnitude and direction of metabolic adjustments relative to control conditions. This figure captures the distinct temporal orchestration of metabolic responses between tissues under osmotic stress, highlighting the sophisticated regulation of both broad chemical classes and specific functional groups during stress adaptation.


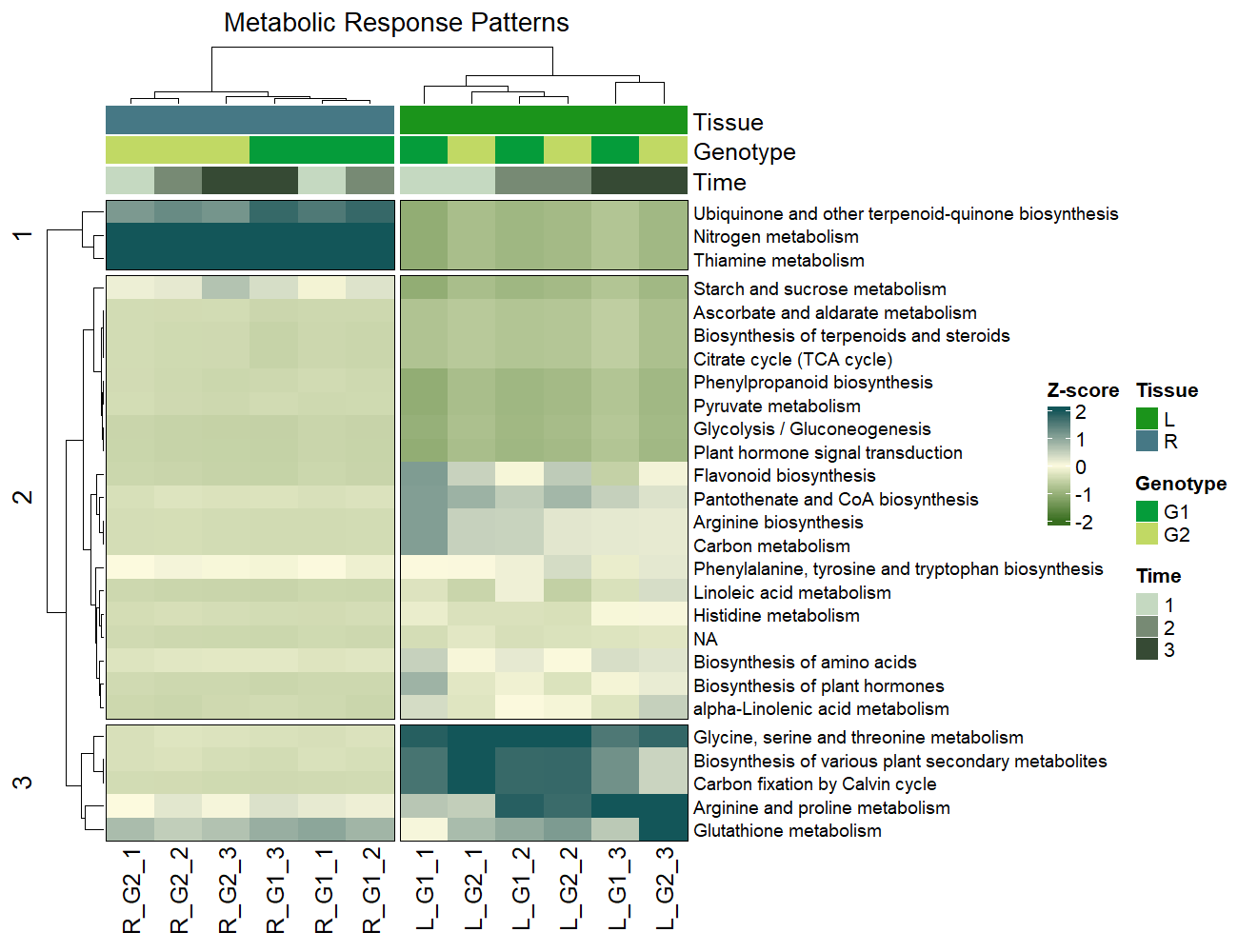


Fig. S4. Tissue and Genotype-Specific Metabolic Pathway Responses under Osmotic Stress in Wheat. Supports Fig. 3: resolves the pathway-level responses underlying the feature dynamics reported in the main text.

This hierarchically clustered heatmap shows Z-score transformed metabolic pathway responses (blue: upregulation, green: downregulation) in leaf (L) and root (R) tissues of drought-tolerant (G1) and drought-susceptible (G2) genotypes across three time points under osmotic stress. Three clusters emerge: (1) downregulated primary metabolism (e.g., ubiquinone biosynthesis), (2) moderately modulated central carbon and amino acid pathways (e.g., TCA cycle), and (3) differential regulation of stress-associated pathways (e.g., glutathione metabolism, flavonoid biosynthesis). The clustering patterns suggest distinct metabolic responses between tissues and genotypes during osmotic stress adaptation.


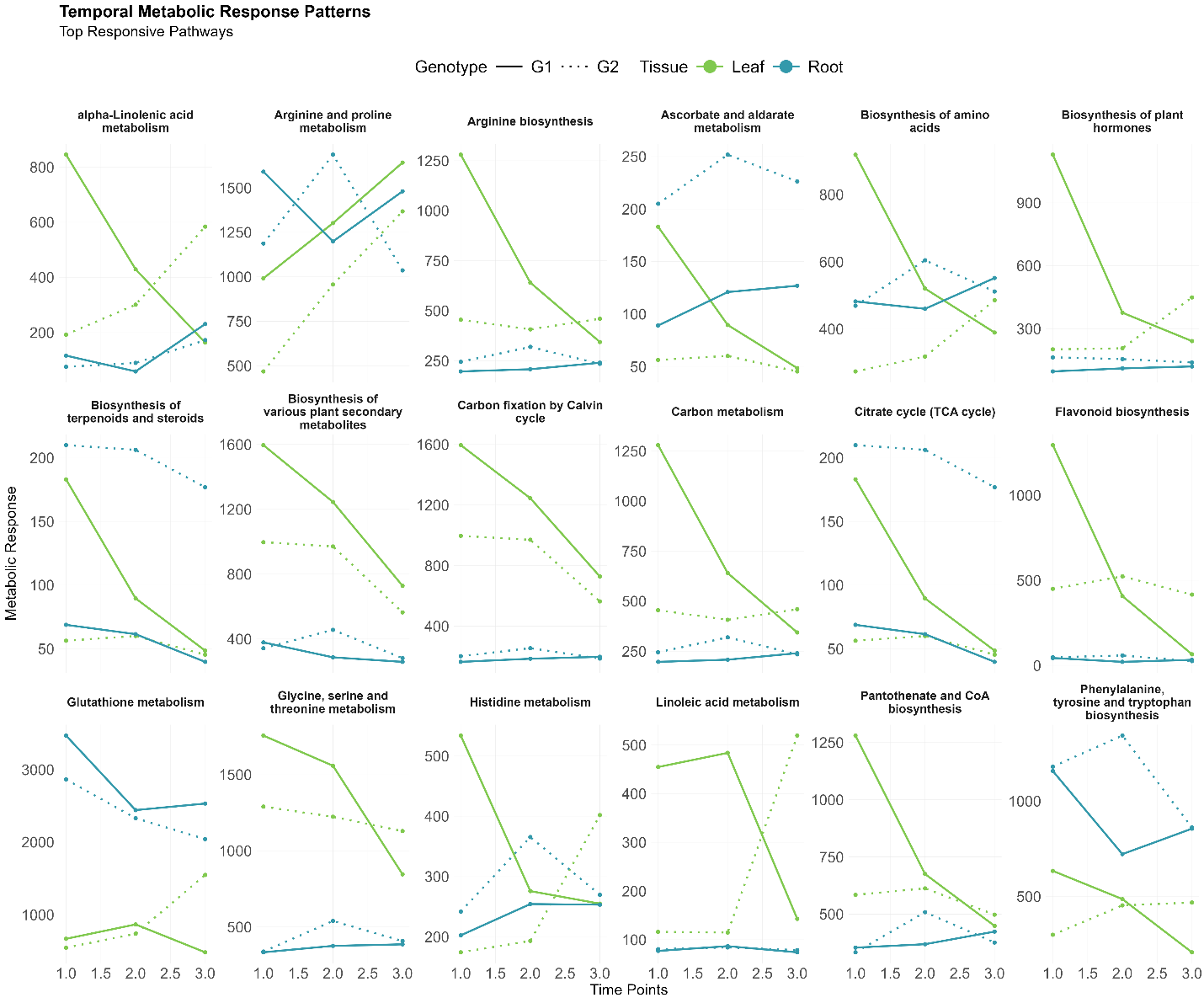


Fig. S5. Temporal Dynamics of Major Metabolic Pathways Under Osmotic Stress. Supports Fig. 3: details the temporal pathway trajectories that contextualise the tissue-specific dynamics in the main text.

Description: Each subplot illustrates the temporal dynamics of putatively identified metabolic pathways in leaf and root tissues under osmotic stress conditions, differentiated by genotype (G1, G2) and tissue type (leaf, root). The visualisation captures tissue-specific response patterns, with leaves typically showing pronounced metabolic adjustments while roots display more gradual changes over time. This aligns with the observed tissue-specific network architecture differences, particularly in G1, where leaves demonstrate coordinated responses across primary metabolism pathways (amino acid biosynthesis, carbon fixation), while roots exhibit modulated changes in stress-responsive pathways (e.g., glutathione metabolism). These pathway-level responses provide additional context for understanding the tissue-specific molecular strategies employed under osmotic stress conditions.


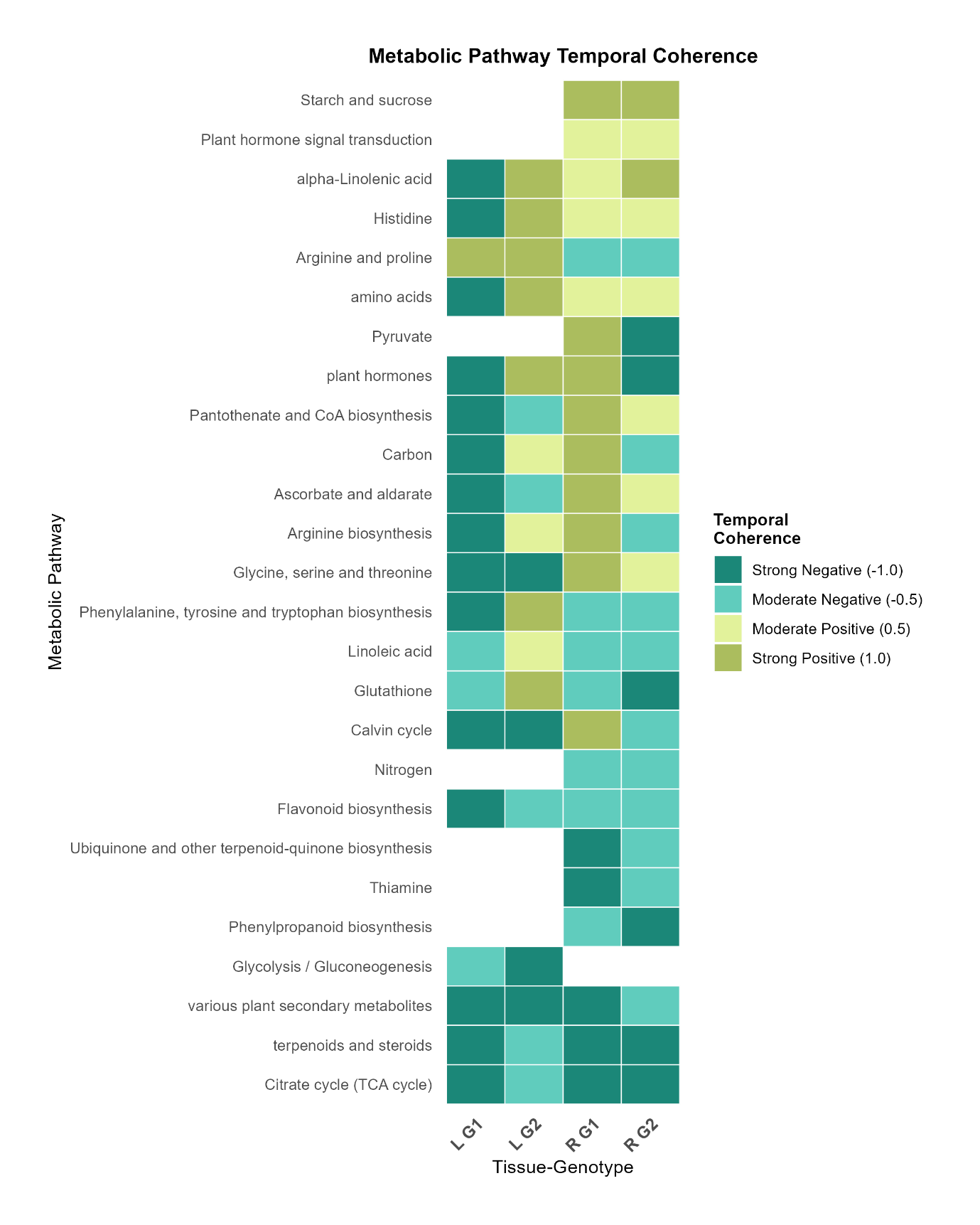


Fig. S6. Tissue-Specific Temporal Coherence of Metabolic Pathways Under Osmotic Stress. Supports Fig. 3: quantifies pathway-level temporal coherence underlying the resilience and dynamism contrasts in the main text.

Description: Heatmap visualisation reveals the temporal coherence patterns of putatively identified metabolic pathways in leaf (L) and root (R) tissues of drought-tolerant (G1) and drought-susceptible (G2) wheat genotypes under osmotic stress. Temporal coherence scores (-1.0 to +1.0) indicate the consistency and direction of metabolic responses over time, where strong positive values (light green) represent sustained increases and strong negative values (dark green) indicate consistent decreases. The analysis captures tissue-specific regulation of multiple pathways, including primary metabolism (amino acids, carbon metabolism) and stress-responsive processes (glutathione, flavonoid biosynthesis). These pathway-level temporal coherence patterns provide additional context for understanding the metabolic responses under osmotic stress conditions.


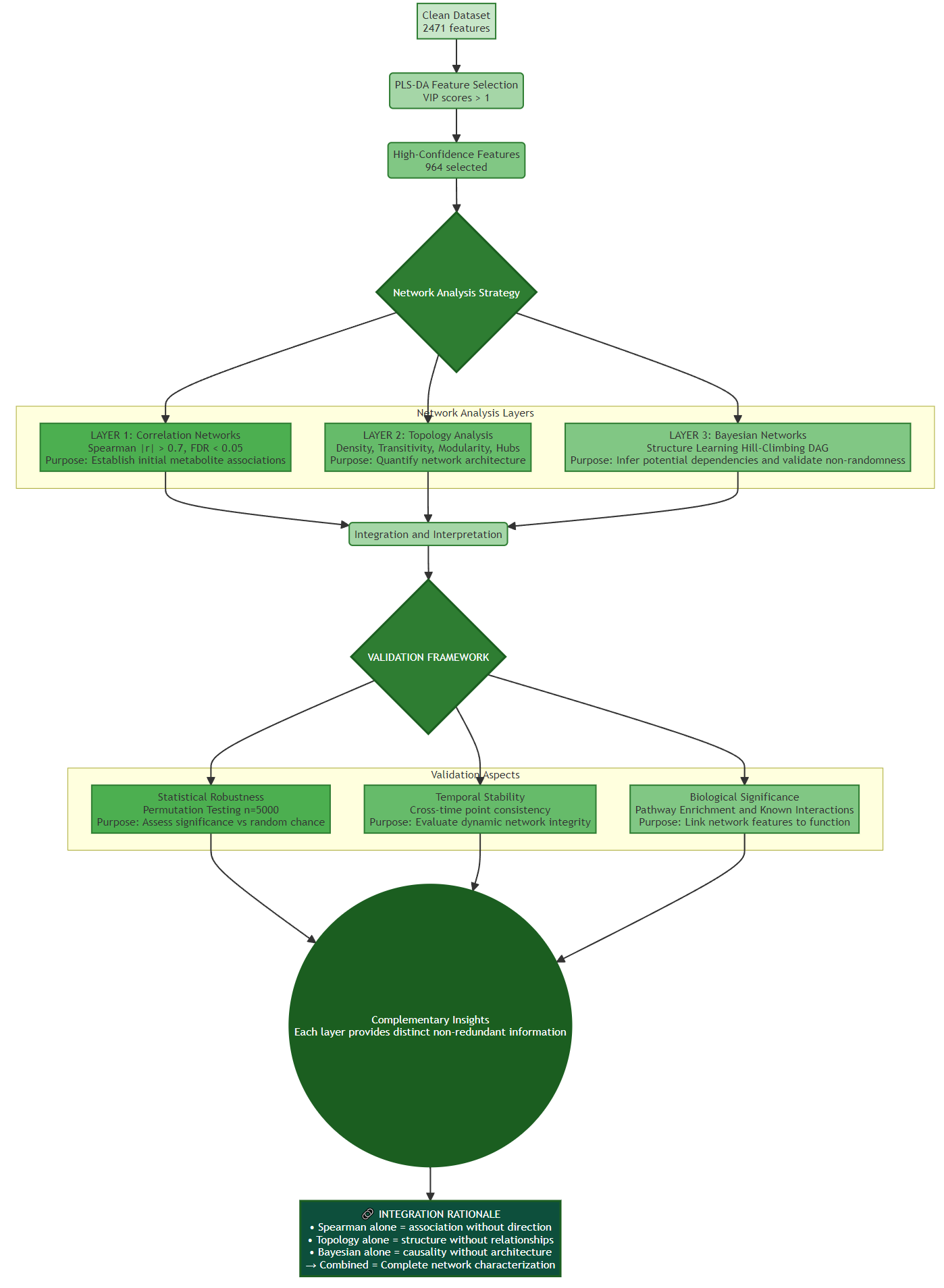


Fig. S7. Multi-layered analytical framework for metabolomic correlation-network analysis. Supports Figs. 1 and 4: outlines the layered analytical framework applied throughout the main-text network analyses.

Each layer addresses distinct biological questions using complementary statistical approaches. Layer 1 identifies co-regulated metabolite pairs, Layer 2 quantifies architectural principles distinguishing tissue-specific organisation strategies, and Layer 3 provides a complementary conditional-dependence representation under directed-acyclic-graph (DAG) assumptions. Statistical robustness was assessed through bootstrap resampling (n = 5,000) and permutation testing, with the number of iterations ranging from 1,000 to 10,000 according to the analysis.


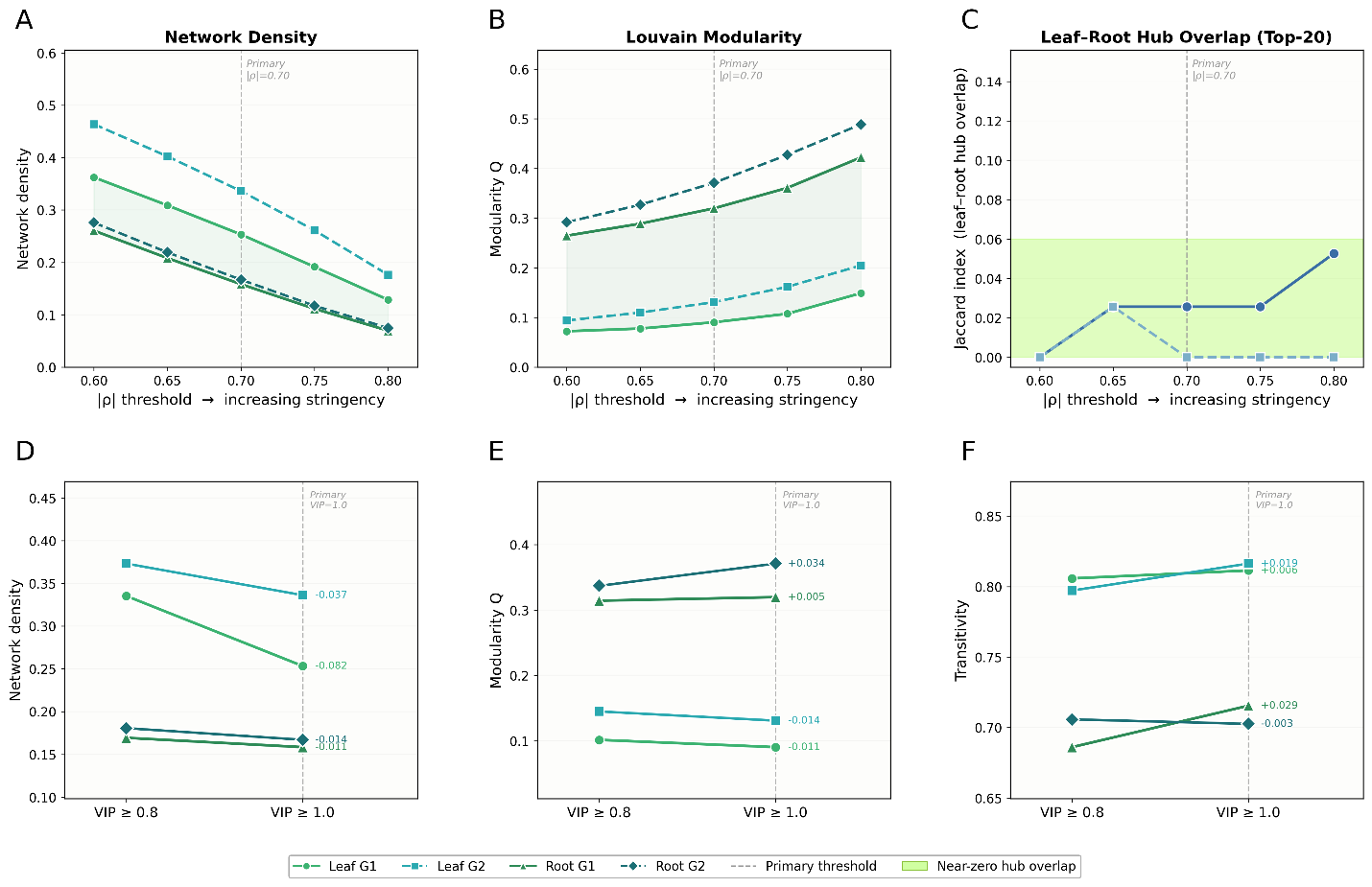


Fig. S8. Leaf–root metabolomic correlation-network architecture is robust to threshold choices. Supports Fig. 1 and Fig. 4: confirms the leaf-denser/root-more-modular asymmetry is invariant to correlation and VIP thresholds.

(A–C) Using stressed samples (Treatment = 1), networks were reconstructed across five absolute Spearman thresholds (|ρ| = 0.60–0.80; BH-FDR < 0.05) at VIP ≥ 1.0. Across the full stringency range, the qualitative architecture is preserved: leaf networks remain denser, root networks remain more modular, and leaf–root hub overlap (top-20; Jaccard) stays near zero, indicating tissue-specific hub identity. (D–F) At the primary correlation threshold (|ρ| = 0.70), relaxing feature selection to VIP ≥ 0.8 changes network size but does not alter the same leaf–root asymmetry in density, modularity, and transitivity. Dashed lines mark the primary thresholds; Δ labels in D–F report VIP ≥ 1.0 minus VIP ≥ 0.8. Underlying values are provided in Supplementary Tables S4–S5.


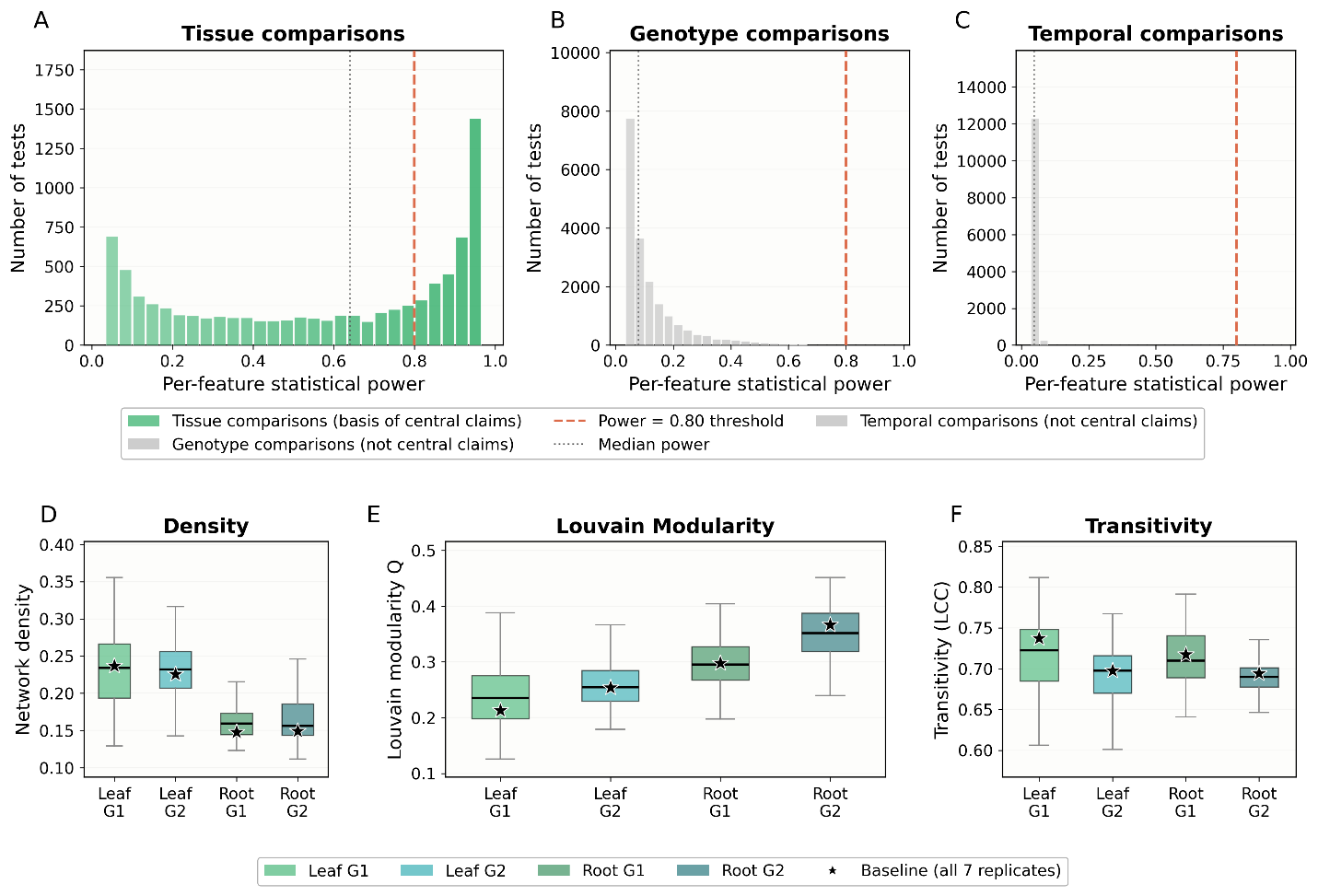


Fig. S9. Network metric stability and per-feature statistical power across comparison types. Supports Fig. 1: confirms network-metric stability and reports per-feature statistical power at n = 7.

(A–C) Distributions of per-feature statistical power for the non-parametric comparisons used in this study: tissue (A; basis of central claims), genotype (B), and temporal (C). The dashed line marks the conventional power threshold (0.80) and the dotted line marks the median. (D–F) Cluster-bootstrap resampling by biological replicate was used to quantify stability of network metrics (density, Louvain modularity Q, and transitivity of the largest connected component) for each tissue × genotype network. Boxplots show bootstrap distributions; stars mark baseline estimates computed using all 7 replicates. Baseline values fall within the bootstrap interquartile range, indicating that network metrics are stable to replicate composition. Underlying values are provided in Supplementary Tables S6 and S7.


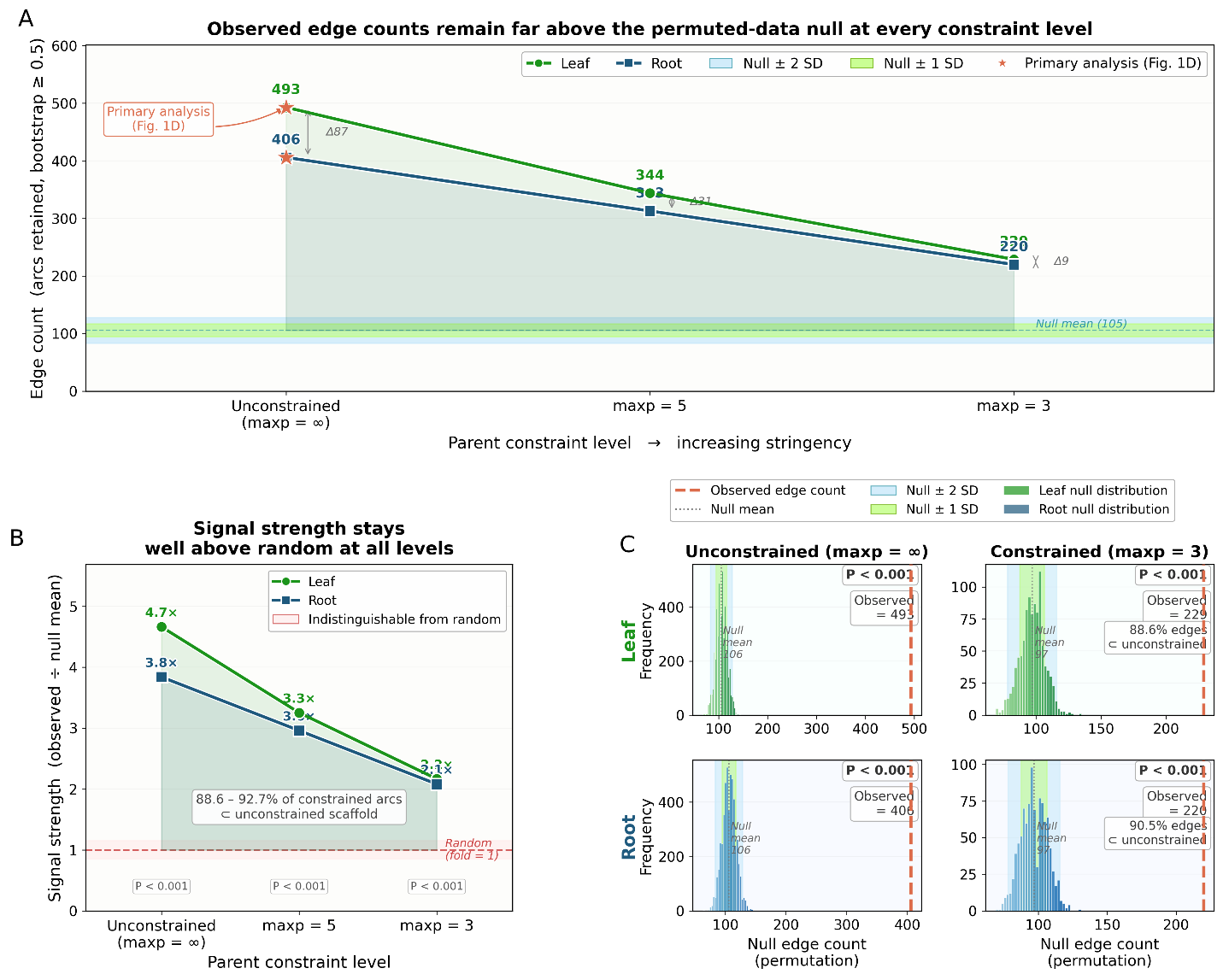


Fig. S10. Bayesian network conclusions are robust to maximum-parent (maxp) constraints. Supports Fig. 1D and Fig. 4: confirms the Bayesian layer’s arc retention and leaf–root ordering across constraint regimes.

Hill-climbing Bayesian structure learning was repeated under three maximum-parent regimes (unconstrained, maxp = 5, maxp = 3), retaining arcs with bootstrap strength ≥ 0.5 and benchmarking against a permuted-data null. (A) Observed arc counts for leaf and root remain far above the permuted-data null at every constraint level; counts decrease monotonically with increasing stringency while the leaf–root ordering is preserved. (B) Signal strength (observed arcs relative to the null mean) remains well above random under all constraints, and constrained networks retain a large subset of arcs from the unconstrained scaffold, indicating that maxp primarily prunes additional arcs rather than altering the dependency backbone. (C) Null distributions for unconstrained versus maxp = 3 show observed arc counts in the extreme tail for both tissues (P < 0.001), confirming non-random structure even under stringent parent caps. Underlying summary values are provided in Supplementary Table S10.

# REFERENCES

**Albert, R., Jeong, H., and Barabási, A.-L.** (2000). Error and attack tolerance of complex networks. Nature **406,** 378-382.

**Alon, U.** (2007). Network motifs: theory and experimental approaches. Nature Reviews Genetics **8,** 450-461.

**Barabási, A.-L., and Albert, R.** (1999). Emergence of Scaling in Random Networks. Science **286,** 509-512.

**Barabási, A.-L., and Oltvai, Z.N.** (2004). Network biology: understanding the cell's functional organization. Nature Reviews Genetics **5,** 101-113.

**Clauset, A., Shalizi, C.R., and Newman, M.E.** (2009). Power-law distributions in empirical data. SIAM review **51,** 661-703.

**Erdos, P., and Rényi, A.** (1960). On the evolution of random graphs. Publ. math. inst. hung. acad. sci **5,** 17-60.

**Fait, A., Batushansky, A., Shrestha, V., Yobi, A., and Angelovici, R.** (2020). Can metabolic tightening and expansion of co-expression network play a role in stress response and tolerance? Plant Science **293,** 110409.

**Fortunato, S.** (2010). Community detection in graphs. Physics reports **486,** 75-174.

**Gargallo-Garriga, A., Sardans, J., Pérez-Trujillo, M., Rivas-Ubach, A., Oravec, M., Vecerova, K., Urban, O., Jentsch, A., Kreyling, J., Beierkuhnlein, C., Parella, T., and Peñuelas, J.** (2014). Opposite metabolic responses of shoots and roots to drought. Scientific Reports **4,** 6829.

**Girvan, M., and Newman, M.E.J.** (2002). Community structure in social and biological networks. Proceedings of the National Academy of Sciences **99,** 7821-7826.

**Grafahrend-Belau, E., Junker, B.H., Klukas, C., Koschützki, D., Schreiber, F., and Schwöbbermeyer, H.** (2009). Topology of plant metabolic networks. Plant metabolic networks**,** 173-209.

**Jeong, H., Mason, S.P., Barabási, A.-L., and Oltvai, Z.N.** (2001). Lethality and centrality in protein networks. Nature **411,** 41-42.

**Karpievitch, Y.V., Dabney, A.R., and Smith, R.D.** (2012). Normalization and missing value imputation for label-free LC-MS analysis. BMC Bioinformatics **13,** S5.

**Kokla, M., Virtanen, J., Kolehmainen, M., Paananen, J., and Hanhineva, K.** (2019). Random forest-based imputation outperforms other methods for imputing LC-MS metabolomics data: a comparative study. BMC Bioinformatics **20**.

**Langfelder, P., Luo, R., Oldham, M.C., and Horvath, S.** (2011). Is my network module preserved and reproducible? PLoS computational biology **7,** e1001057.

**Latora, V., and Marchiori, M.** (2001). Efficient behavior of small-world networks. Physical review letters **87,** 198701.

**Newman, M.** (2010). Networks: An Introduction. (Oxford University Press).

**Newman, M.E.** (2002). Assortative mixing in networks. Physical review letters **89,** 208701.

**Newman, M.E.J.** (2006). Modularity and community structure in networks. Proceedings of the National Academy of Sciences **103,** 8577-8582.

**Watts, D.J., and Strogatz, S.H.** (1998). Collective dynamics of ‘small-world’ networks. Nature **393,** 440-442.

**Wei, R., Wang, J., Su, M., Jia, E., Chen, S., Chen, T., and Ni, Y.** (2018). Missing value imputation approach for mass spectrometry-based metabolomics data. Scientific reports **8,** 663.

**Zhang, B., and Horvath, S.** (2005). A general framework for weighted gene co-expression network analysis. Stat Appl Genet Mol Biol **4,** Article17.
